# Gut microbiota influences lung cancer risk through circulating cytokines—Insights from a Mendelian randomization study

**DOI:** 10.1097/MD.0000000000044897

**Published:** 2025-10-03

**Authors:** Wei Shen, Ruolin Hou, Jianjie Zhang, Chengyi Liu, Changzhen Zhang, Xin Liu

**Affiliations:** a The Third People’s Hospital Health Care Group of Cixi, Ningbo, China; b Jiangxi University of Chinese Medicine, Nanchang, China; c Ningbo No. 2 Hospital, Ningbo, China.

**Keywords:** causal effect, circulating cytokines, lung adenocarcinoma, lung squamous cell carcinoma, mediation analysis, small cell lung cancer

## Abstract

Lung cancer is the leading cause of cancer-related deaths worldwide. Gut microbiota and circulating cytokines may play important roles in the development and progression of lung cancer, but their causality has not been clarified. In this study, we comprehensively assessed the causal associations between 473 types of gut microbiota and 91 types of circulating cytokines with 3 types of lung cancers, including squamous cell carcinoma, adenocarcinoma, and small cell lung cancer, by using Mendelian randomization analysis in conjunction with the most recent genome-wide association studies data and lung cancer cohort data. The mediating role of circulating cytokines in the relationship between gut microbiota and lung cancer was further explored using mediation analyses. Mediation analysis identified 6 important circulating cytokines that intricately regulate the development and progression of 3 types of lung cancer. Lung squamous carcinoma was more susceptible to circulating cytokines than lung adenocarcinoma and small cell lung cancer. In this study, we constructed a causal network of “gut microbiota-circulating cytokines-lung cancer” at a multi-omics level, and revealed the causal relationship between specific gut microbiota and circulating cytokines in the development and progression of lung cancer, which will provide new targets and strategies for the prevention and treatment of lung cancer in the future.

## 1. Introduction

Lung cancer is the leading cause of cancer-related deaths worldwide, with a complex pathogenesis and poor prognosis.^[1]^ Global cancer statistics for 2021 show that there are approximately 2.3 million new cases of lung cancer and 1.8 million deaths, accounting for 18% of all cancer deaths, and the 5-year survival rate for advanced patients is still <20%.^[2]^ Lung cancer mainly includes lung squamous cell carcinoma (LSCC), lung adenocarcinoma (LUAD), and small cell lung cancer (SCLC) in histologic subtype classification, which together account for approximately 80% of all lung cancer cases.^[3,4]^ Although targeted therapies and immune checkpoint inhibitors have significantly improved the survival of some patients, drug resistance, tumor heterogeneity, and low response rates to immunotherapy remain major clinical challenges.^[5,6]^ In addition, the efficacy of chemotherapy and radiotherapy is approaching a plateau, and there is an urgent need to explore novel targets for intervention from the perspective of host-microenvironment interactions.

In recent years, the role of gut microbiota and circulating cytokines in lung cancer has gradually become a research hotspot. Observational studies have shown that dysregulation of gut microbiota may affect the lung immune microenvironment by influencing the metabolism of short-chain fatty acids or lipopolysaccharides, thereby affecting the occurrence and progression of lung cancer.^[7,8]^ Moreover, changes in the levels of circulating cytokines, which are important markers of inflammatory responses, are closely related to the prognosis of lung cancer patients.^[9,10]^ However, there are still limitations in the current studies on the causal relationship between gut microbiota and circulating cytokines in lung cancer, mainly in that most of the studies are limited to observational studies, and confounding factors are difficult to exclude from traditional observational studies, making it difficult to determine the causal relationship.^[11,12]^ In addition, the causal pathway of how gut microbiota affects lung cancer by modulating cytokine networks remains unclear, limiting its clinical translational potential. Therefore, conducting causality studies is crucial to unravel the mechanisms of gut microbiota and circulating cytokines in lung cancer.

Mendelian randomization (MR), which utilizes genetic variation as an instrumental variable (IV), can effectively circumvent the confounding bias of traditional observational studies and has become a central method for causal inference in recent years.^[13]^ In microbiome studies, large-scale genome-wide association studies (GWAS) have identified genetic loci significantly associated with gut microbiota abundance (e.g., MiBioGen consortium data), providing a reliable tool for MR analysis.^[14,15]^ Further, MR mediation analysis, by decomposing the total effect of exposure on outcome into direct and mediated effects, is capable of effectively elucidating the causal chain of multi-omics factors,^[16]^ which may provide new ideas for the prevention and treatment of complex diseases.

Based on the above background, this study intends to comprehensively and systematically evaluate the causal associations between 473 types of gut microbiota and 91 types of circulating cytokines with LSCC, adenocarcinoma, and SCLC in conjunction with the most recent GWAS data, and to explore the differences in the roles of gut microbiota and cytokines in different lung cancer subtypes. This study will construct a causal network of “gut microbiota-circulating cytokines-lung cancer” at a multi-omics level, and the results will not only help to further understand the pathogenesis of lung cancer, but also provide a scientific basis for future intervention strategies targeting gut microbiota and circulating cytokines.

## 2. Methods

### 2.1. Study design

**Figure 1.**
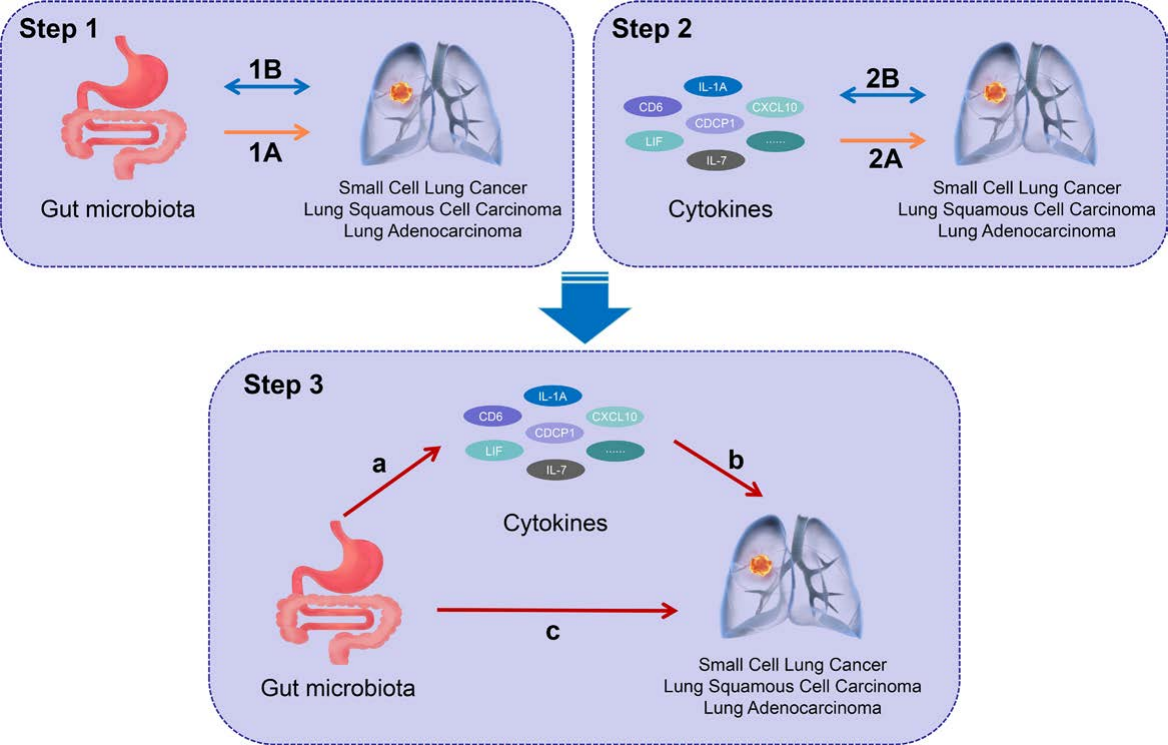
Study overview. Step 1A: Parse the causal association of gut microbiota on lung carcinogenesis. Step 1B: Examine bidirectional causal associations between gut microbiota and lung cancer. Step 2A: Analyze the causal association of cytokines on lung cancer. Step 2B: Test the bidirectional causal association between lung cancer on cytokines. Step 3: Mediation analysis of cytokines in the pathway from gut microbiota to lung cancer. Pathway a: The causal effect of gut microbiota on cytokines. Pathway b: The causal effect of cytokines on lung cancer. Pathway c: The total effect of gut microbiota on lung cancer.

This study contains 3 main parts, as shown in Figure 1: first, we explored the causal effects of 473 types of gut microbiota on lung cancer (Step 1A); second, we analyzed the causal effects of 91 types of cytokines on lung cancer (Step 2A); and lastly, we performed an analysis of the mediating effects of cytokines in the pathway from gut microbiota to lung cancer (Step 3). We used single nucleotide polymorphisms (SNPs) as IVs. The MR approach relies on 3 key assumptions: that there is a strong association between the IV and the exposure factor; the IV is independent of potential confounders; and the IV does not have a direct effect on the outcome, but rather affects the outcome only indirectly through the exposure factor.^[17,18]^

### 2.2. Data source

Gut microbiota GWAS data: Comprehensive summary statistics for gut microbiota, including genome-wide significant results, are publicly available in the NHGRI-EBI GWAS Catalog (ranging from accession GCST90032172 to GCST90032644.1). Genetic variants linked to the gut microbiome were identified through GWAS involving 7,979,834 human genetic variants from 5959 individuals in the FINRISK 2002 cohort. The analysis revealed 567 independent SNP-taxon associations related to gut microbial taxa.^[14]^ Cytokine levels were derived from a recent investigation encompassing 11 cohorts, with a total of 14,824 participants of European descent. The specific study design and data control processes have been documented in previous reports.^[19]^ The levels of all 91 cytokines were assessed via the Olink Target Inflammation panel, and genome-wide genetic information was also obtained.

Considering the parameters of sample size, sequencing depth, ethnicity consistency, and data update time, the 3 types of lung cancer datasets in this study were from Finngen version 12, in which LSCC included 1898 cases (gs://finngen-public-data-r12/summary_stats/release/finngen_R12_C3_NSCLC_SQUAM_EXALLC.gz), LUAD included 2219 cases (gs://finngen-public-data-r12/summary_stats/release/finngen_R12_C3_NSCLC_ADENO_EXALLC.gz), and SCLC included 909 cases (gs://finngen-public-data-r12/summary_stats/release/finngen_R12_C3_SCLC_EXALLC.gz), and control 378,749 cases.

The GWAS data for the aforementioned exposures and outcomes were all sourced from independent cohorts with no sample overlap. This study utilized publicly available GWAS pooled data for secondary analysis. All original GWAS studies were ethically reviewed. In addition, this study did not utilize any individual-level data and therefore did not require additional ethics committee approval.

### 2.3. IVs selection

Initially, we selected the SNPs that exhibited strong associations with gut microbiota and circulating cytokines (*P* < 1 × 10^−6^). To ensure a sufficient number of IVs for different lung cancer types, we chose SNPs with a *P*-value threshold of 5 × 10^−5^. Subsequently, we removed SNPs with linkage disequilibrium from the analysis. The selected SNPs strongly associated with gut microbiota had to satisfy the criteria of *r*^2^ < 0.001 and distance >10,000 kb. Following the outcome matching, we eliminated palindromic SNPs.

We gathered the essential data, including chromosome, effect allele, other allele, effect allele frequency (EAF), effect sizes (β), standard error (SE), and *P*-value. Subsequently, we determined the explained variance (*R*^2^) and *F*-statistic to evaluate if the identified IVs were strongly linked to the exposure. Generally, SNPs with an *F*-statistic below 10 are regarded as weak instruments.^[20]^ In this study, *R*^2^ was calculated as 2 × EAF × (1 − EAF) × β^2^ divided by [2 × EAF × (1 − EAF) × β^2^ + 2 × EAF × (1 − EAF) × N × SE^2^], where N represents the sample size of the GWAS for the FI, and the *F*-statistic is given by *F* = *R*^2^(N − 2)/(1 − *R*^2^).

### 2.4. MR analysis

#### 2.4.1. Primary analysis

To assess the causal impact of gut microbiota and cytokines on lung cancer, we conducted a 2-sample MR analysis for each. The inverse variance weighted (IVW) method served as the primary analytical technique.^[21]^ The outcomes of the MR analysis were presented as odds ratios (ORs), accompanied by their respective 95% confidence intervals (CIs). Statistical significance was determined when the *P*-value associated with the IVW method was below .05, and when the direction of the results from both the IVW and MR-Egger methods aligned.

#### 2.4.2. Multiple testing correction

The Benjamini–Hochberg (BH) procedure was employed to conduct false discovery rate (FDR) correction.^[22]^ For each stage, the effective threshold (*P*′) was reestimated according to the number of univariable MR analyses performed in various directions. The formula used is as follows: $P^{\prime }=\left(i/m\right)\alpha$, where “*i*” denotes the rank position of the *P*-value, “*m*” represents the total number of tests conducted, and “α” is the prespecified significance level (α = 0.05). Results with *P* ≥ .05 are not significant; those with *P*′ ≤ *P* < .05 are regarded as nominally significant; and those with *P* < *P*′ are considered significant.

#### 2.4.3. Mediation analysis

The above gut microbiota and cytokines with significant causal effects on lung cancer were included in the mediation analysis. We investigated whether gut microbiota exerted a causal influence on cytokines. If such an effect was identified, we planned to conduct multiple MR analyses to determine if cytokines acted as mediating factors in the pathway from gut microbiota to lung cancer.

#### 2.4.4. Bidirectional causality analysis

To examine the reverse causality between gut microbiota, circulating cytokines, and lung cancer, we performed reverse MR analysis. Notably, when conducting mediation analysis, it is essential to exclude these reverse causal effects to avoid reversed causation. We designated lung cancer as the “exposure” and gut microbiota or cytokines linked to lung cancer as the “outcome.” For IVs, we chose SNPs that exhibited a significant association with lung cancer (*P* < 5 × 10^−5^).

### 2.5. Sensitivity analysis

We conducted Cochran *Q* test to assess the heterogeneity of each SNP and created scatter plots to illustrate SNP-exposure associations and SNP-outcome associations, thereby visualizing the MR results. Leave-one-out analysis was carried out to determine whether each SNP could influence the results. Additionally, we employed MR-PRESSO and MR-Egger regression to examine potential horizontal pleiotropy effects. MR-PRESSO was utilized to identify significant outliers and to adjust for horizontal pleiotropy by eliminating these outliers.

All data analysis packages employed were obtained from the open-access repository in the R software platform (version 4.3.2; R Development Core Team).

## 3. Results

### 3.1. Selection of IVs

First, we identified 5259 SNPs linked to 473 types of gut microbiota with a significance threshold of *P* < 1 × 10^−6^. These 5259 SNPs were then designated as IVs for the gut microbiota taxa (Table S1, Supplemental Digital Content, https://links.lww.com/MD/Q195). Subsequently, we detected 1818 SNPs that were significantly associated with 91 cytokines with a significance threshold of *P* < 1 × 10^−6^ (Table S2, Supplemental Digital Content, https://links.lww.com/MD/Q195). In the reverse MR study, 101 SNPs were identified when focusing on LSCC (Table S3, Supplemental Digital Content, https://links.lww.com/MD/Q195). When LUAD was the focus of the study, 102 SNPs were identified (Table S4, Supplemental Digital Content, https://links.lww.com/MD/Q195). When the study focused on SCLC, 73 SNPs were identified (Table S5, Supplemental Digital Content, https://links.lww.com/MD/Q195).

### 3.2. MR results of gut microbiota on lung cancer

**Figure 2.**
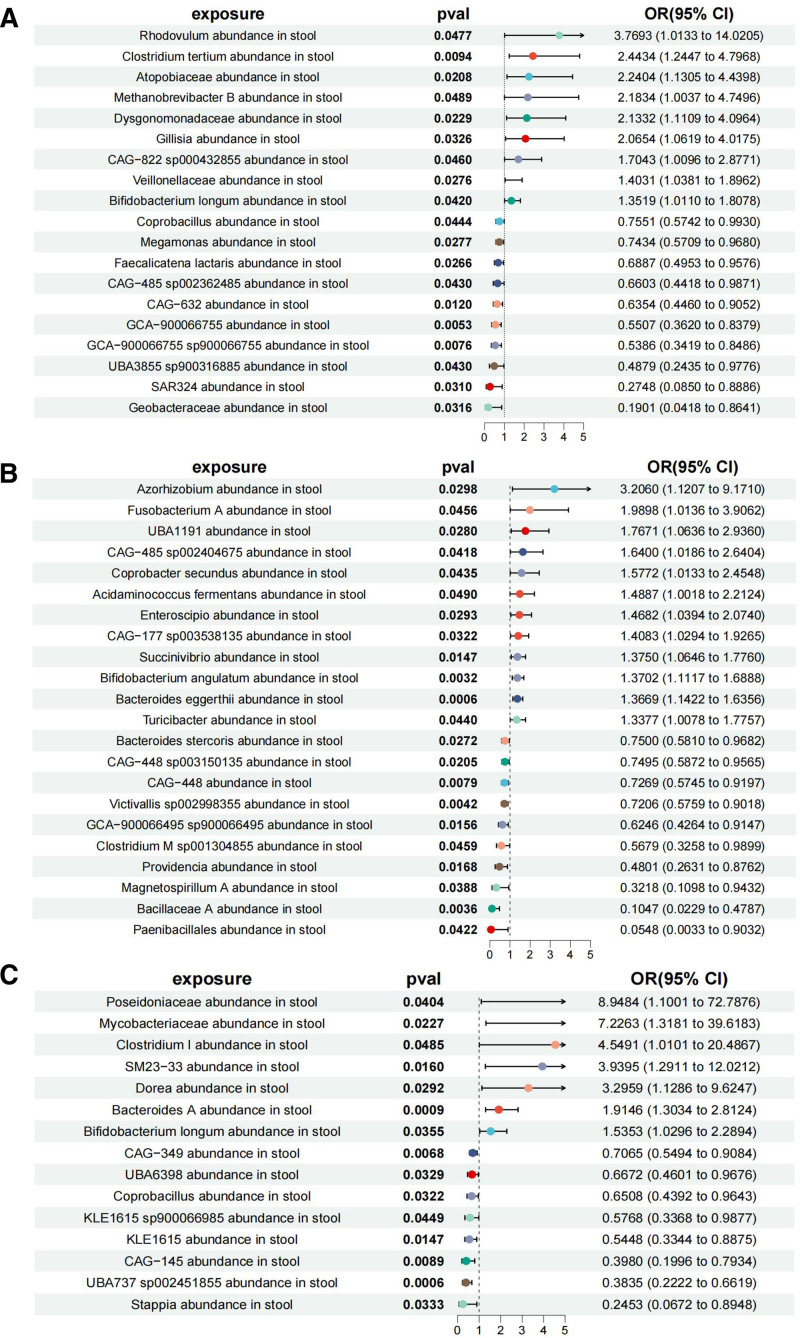
The forest plot of MR analyses between gut microbiota and lung cancer. Causal association between gut microbiota on (A) lung squamous cell carcinoma, (B) lung adenocarcinoma, and (C) small-cell lung cancer. CI = confidence interval, MR = Mendelian randomization, OR = odds ratio.

We used IVW as the primary method. The associated bacterial taxon was identified using GTDB release 89 nomenclature. In our positive MR analysis, 9 types of gut microbiota may increase the risk of LSCC (Table S6, Supplemental Digital Content, https://links.lww.com/MD/Q195, Fig. 2A). *Rhodovulum* abundance in stool (OR = 3.7693, 95% CI: 1.0133–14.0205, IVW: *P* = .0477); *Clostridium tertium* abundance in stool (OR = 2.4434, 95% CI: 1.2447–4.7968, IVW: *P* = .0094); Atopobiaceae abundance in stool (OR = 2.2404, 95% CI: 1.1305–4.4398, IVW: *P* = .0208); *Methanobrevibacter* B abundance in stool (OR = 2.1834, 95% CI: 1.0037–4.7496, IVW: *P* = .0489); Dysgonomonadaceae abundance in stool (OR = 2.1332, 95% CI: 1.1109–4.0964, IVW: *P* = .0229); *Gillisia* abundance in stool (OR = 2.0654, 95% CI: 1.0619–4.0175, IVW: *P* = .0326); CAG-822 sp000432855 abundance in stool (OR = 1.7043, 95% CI: 1.0096–2.8771, IVW. *P* = .0460); Veillonellaceae abundance in stool (OR = 1.4031, 95% CI: 1.0381–1.8962, IVW: *P* = .0276); *Bifidobacterium longum* abundance in stool (OR = 1.3519, 95% CI: 1.0110–1.8078, IVW: *P* = .0420).

There are 12 types of gut microbiota that may increase the risk of LUAD (Table S6, Supplemental Digital Content, https://links.lww.com/MD/Q195, Fig. 2B). *Azorhizobium* abundance in stool (OR = 3.2060, 95% CI: 1.1207–9.1710, IVW: *P* = .0298); *Fusobacterium* A abundance in stool (OR = 1.9898, 95% CI: 1.0136–3.9062, IVW: *P* = .0456); UBA1191 abundance in stool (OR = 1.7671, 95% CI: 1.0636–2.9360, IVW: *P* = .0280); CAG-485 sp002404675 abundance in stool (OR = 1.6400, 95% CI: 1.0186–2.6404, IVW: *P* = .0418); *Coprobacter secundus* abundance in stool (OR = 1.5772, 95% CI: 1.0133–2.4548, IVW: *P* = .0435); *Acidaminococcus fermentans* abundance in stool (OR = 1.4887, 95% CI: 1.0018–2.2124, IVW: *P* = .0490); *Enteroscipio* abundance in stool (OR = 1.4682, 95% CI: 1.0394–2.0740, IVW: *P* = .0293); CAG-177 sp003538135 abundance in stool (OR = 1.4083, 95% CI: 1.0294–1.9265, IVW: *P* = .0322); *Succinivibrio* abundance in stool (OR = 1.3750, 95% CI: 1.0646–1.7760, IVW: *P* = .0147); *Bifidobacterium angulatum* abundance in stool (OR = 1.3702, 95% CI: 1.1117–1.6888, IVW: *P* = .0032); *Bacteroides eggerthii* abundance in stool (OR = 1.3669, 95% CI: 1.1422–1.6356, IVW: *P* = .0006); *Turicibacter* abundance in stool (OR = 1.3377, 95% CI: 1.0078–1.7757, IVW: *P* = .0440).

There are 7 types of gut microbiota that may increase the risk of SCLC (Table S6, Supplemental Digital Content, https://links.lww.com/MD/Q195, Fig. 2C). Poseidoniaceae abundance in stool (OR = 8.9484, 95% CI: 1.1001–72.7876, IVW: *P* = .0404); Mycobacteriaceae abundance in stool (OR = 7.2263, 95% CI: 1.3181–39.6183, IVW: *P* = .0227); *Clostridium* I abundance in stool (OR = 4.5491, 95% CI: 1.0101–20.4867, IVW: *P* = .0485); SM23-33 abundance in stool (OR = 3.9395, 95% CI: 1.2911–12.0212, IVW: *P* = .0160); *Dorea* abundance in stool (OR = 3.2959, 95% CI: 1.1286–9.6247, IVW: *P* = .0292); *Bacteroides* A abundance in stool (OR = 1.9146, 95% CI: 1.3034–2.8124, IVW: *P* = .0009); *Bifidobacterium longum* abundance in stool (OR = 1.5353, 95% CI: 1.0296–2.2894, IVW: *P* = .0355).

On the contrary, 10 types of gut microbiota were protective against LSCC (Table S6, Supplemental Digital Content, https://links.lww.com/MD/Q195, Fig. 2A). *Coprobacillus* abundance in stool (OR = 0.7551, 95% CI: 0.5742–0.9930, IVW: *P* = .0444); *Megamonas* abundance in stool (OR = 0.7434, 95% CI: 0.5709–0.9680, IVW: *P* = .0277); *Faecalicatena lactaris* abundance in stool (OR = 0.6887, 95% CI: 0.4953–0.9576, IVW: *P* = .0266); CAG-485 sp002362485 abundance in stool (OR = 0.6603, 95% CI: 0.4418–0.9871, IVW: *P* = .0430); CAG-632 abundance in stool (OR = 0.6354, 95% CI: 0.4460–0.9052, IVW: *P* = .0120); GCA-900066755 abundance in stool (OR = 0.5507, 95% CI: 0.3620–0.8379, IVW: *P* = .0053); GCA-900066755 sp900066755 abundance in stool (OR = 0.5386, 95% CI: 0.3419–0.8486, IVW: *P* = .0076); UBA3855 sp900316885 abundance in stool (OR = 0.4879, 95% CI: 0.2435–0.9776, IVW: *P* = .0430); SAR324 abundance in stool (OR = 0.2748, 95% CI: 0.0850–0.8886, IVW: *P* = .0310); Geobacteraceae abundance in stool (OR = 0.1901, 95% CI: 0.0418–0.8641, IVW: *P* = .0316).

Ten types of gut microbiota were protective against LUAD (Table S6, Supplemental Digital Content, https://links.lww.com/MD/Q195, Fig. 2B). *Bacteroides stercoris* abundance in stool (OR = 0.7500, 95% CI: 0.5810–0.9682, IVW: *P* = .0272); CAG-448 sp003150135 abundance in stool (OR = 0.7495, 95% CI: 0.5872–0.9565, IVW: *P* = .0205); CAG-448 abundance in stool (OR = 0.7269, 95% CI: 0.5745–0.9197, IVW: *P* = .0079); *Victivallis* sp002998355 abundance in stool (OR = 0.7206, 95% CI: 0.5759–0.9018, IVW: *P* = .0042); GCA-900066495 sp900066495 abundance in stool (OR = 0.6246, 95% CI: 0.4264–0.9147, IVW: *P* = .0156); *Clostridium* M sp001304855 abundance in stool (OR = 0.5679, 95% CI: 0.3258–0.9899, IVW: *P* = .0459); *Providencia* abundance in stool (OR = 0.4801, 95% CI: 0.2631–0.8762, IVW: *P* = .0168); *Magnetospirillum* A abundance in stool (OR = 0.3218, 95% CI: 0.1098–0.9432, IVW: *P* = .0388); Bacillaceae A abundance in stool (OR = 0.1047, 95% CI: 0.0229–0.4787, IVW: *P* = .0036); *Paenibacillales* abundance in stool (OR = 0.0548, 95% CI: 0.0033–0.9032, IVW: *P* = .0422).

Eight types of gut microbiota were protective against SCLC (Table S6, Supplemental Digital Content, https://links.lww.com/MD/Q195, Fig. 2C). CAG-349 abundance in stool (OR = 0.7065, 95% CI: 0.5494–0.9084, IVW: *P* = .0068); UBA6398 abundance in stool (OR = 0.6672, 95% CI: 0.4601–0.9676, IVW: *P* = .0329); *Coprobacillus* abundance in stool (OR = 0.6508, 95% CI: 0.4392–0.9643, IVW: *P* = .0322); KLE1615 sp900066985 abundance in stool (OR = 0.5768, 95% CI: 0.3368–0.9877, IVW: *P* = .0449); KLE1615 abundance in stool (OR = 0.5448, 95% CI: 0.3344–0.8875, IVW: *P* = .0147); CAG-145 abundance in stool (OR = 0.3980, 95% CI: 0.1996–0.7934, IVW: *P* = .0089); UBA737 sp002451855 abundance in stool (OR = 0.3835, 95% CI: 0.2222–0.6619, IVW: *P* = .0006); *Stappia* abundance in stool (OR = 0.2453, 95% CI: 0.0672–0.8948, IVW: *P* = .0333).

After the FDR correction using the BH method, the results showed that although *P*-value was <.05, they were ≥*P′*. These results are nominally significant. The overall direction of the effect remains consistent, suggesting it may have potential biological significance. Using the MR-Egger regression intercept method, we determined that genetic pleiotropy did not introduce bias into the results. Additionally, MR-PRESSO analysis confirmed the absence of horizontal pleiotropy within the MR investigation (Table S6, Supplemental Digital Content, https://links.lww.com/MD/Q195). Cochran *Q* tests indicated no significant heterogeneity. The “leave-one-out” analysis demonstrated the reliability of the MR analysis. Furthermore, visual inspection of the funnel and scatter plots revealed no evident asymmetries or heterogeneities (Figures S1–S12, Supplemental Digital Content, https://links.lww.com/MD/Q196).

### 3.3. MR results of cytokines on lung cancer

**Figure 3.**
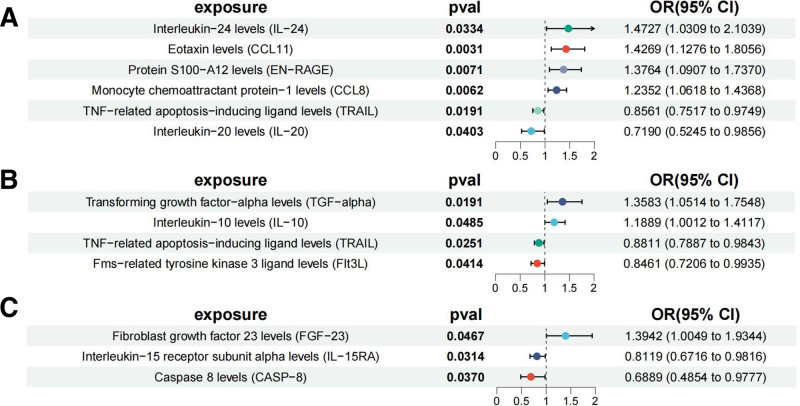
The forest plot of MR analyses between cytokines and lung cancer. Causal association between cytokines on (A) lung squamous cell carcinoma, (B) lung adenocarcinoma, and (C) small-cell lung cancer. CI = confidence interval, MR = Mendelian randomization, OR = odds ratio.

There are 4 types of cytokines that may increase the risk of LSCC (Table S7, Supplemental Digital Content, https://links.lww.com/MD/Q195, Fig. 3A). Interleukin-24 (IL-24) levels (OR = 1.4727, 95% CI: 1.0309–2.1039, IVW: *P* = .0334); Eotaxin levels (CCL11) (OR = 1.4269, 95% CI: 1.1276–1.8056, IVW: *P* = .0031); Protein S100-A12 levels (EN-RAGE) (OR = 1.3764, 95% CI: 1.0907–1.7370, IVW: *P* = .0071); monocyte chemoattractant protein-1 levels (CCL8) (OR = 1.2352, 95% CI: 1.0618–1.4368, IVW: *P* = .0062). Two types of cytokines may increase the risk of LUAD (Table S7, Supplemental Digital Content, https://links.lww.com/MD/Q195, Fig. 3B). Transforming growth factor-alpha (TGF-alpha) levels (OR = 1.3583, 95% CI: 1.0514–1.7548, IVW: *P* = .0191); interleukin-10 (IL-10) levels (OR = 1.1889, 95% CI: 1.0012–1.4117, IVW: *P* = .0485). One cytokine that may increase the risk of SCLC (Table S7, Supplemental Digital Content, https://links.lww.com/MD/Q195, Fig. 3C). Fibroblast growth factor 23 (FGF-23) levels (OR = 1.3942, 95% CI: 1.0049–1.9344, IVW: *P* = .0467).

On the contrary, 2 types of cytokines may reduce the risk of LSCC (Table S7, Supplemental Digital Content, https://links.lww.com/MD/Q195, Fig. 3A). TNF-related apoptosis-inducing ligand (TRAIL) levels (OR = 0.8561, 95% CI: 0.7517–0.9749, IVW: *P* = .0191); interleukin-20 (IL-20) levels (OR = 0.7190, 95% CI: 0.5245–0.9856, IVW: *P* = .0403). Two types of cytokines may reduce the disease risk of LUAD (Table S7, Supplemental Digital Content, https://links.lww.com/MD/Q195, Fig. 3B). TNF-related apoptosis-inducing ligand (TRAIL) levels (OR = 0.8811, 95% CI: 0.7887–0.9843, IVW: *P* = .0251); Fms-related tyrosine kinase 3 ligand (FIt3L) levels (OR = 0.8461, 95% CI: 0.7206–0.9935, IVW: *P* = .0414). Two types of cytokines may reduce the risk of SCLC (Table S7, Supplemental Digital Content, https://links.lww.com/MD/Q195, Fig. 3C). Interleukin-15 receptor subunit alpha (IL-15RA) levels (OR = 0.8119, 95% CI: 0.6716–0.9816, IVW. *P* = .0314); Caspase 8 (CASP-8) levels (OR = 0.6889, 95% CI: 0.4854–0.9777, IVW: *P* = .0370). After the FDR correction using the BH method, the results showed that although *P*-value was <.05, they were ≥*P′*. These results are nominally significant. The overall direction of the effect remains consistent. Sensitivity analyses showed no heterogeneity and pleiotropy, and the reliability of our results was further verified by “leave-one-out” analyses, funnel plots, and scatter plots (Figures S13–S24, Supplemental Digital Content, https://links.lww.com/MD/Q196).

### 3.4. Association of gut microbiota and cytokines

**Figure 4.**
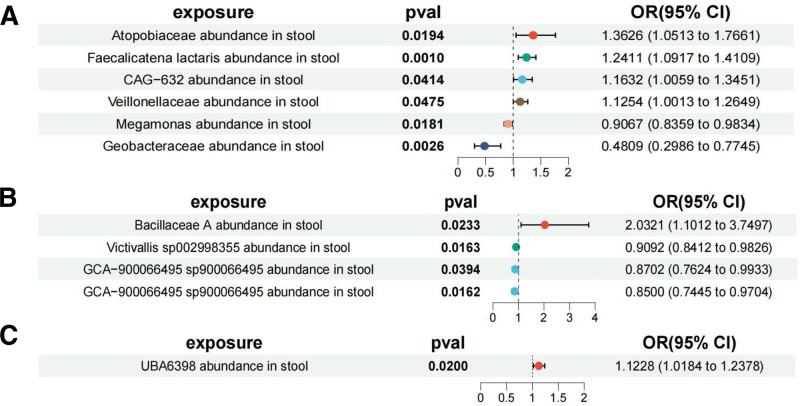
The forest plot of MR analyses between gut microbiota and cytokines: (A) in lung squamous cell carcinoma, (B) in lung adenocarcinoma, and (C) in small-cell lung cancer. CI = confidence interval, MR = Mendelian randomization, OR = odds ratio.

We performed MR analyses of selected gut microbiota and circulating cytokines in the above 3 types of lung cancer. Our analysis yielded several important findings. The same gut microbiota could be significantly associated with multiple cytokines (Table S8, Supplemental Digital Content, https://links.lww.com/MD/Q195), for example, GCA-900066495 sp900066495 abundance in stool (Fig. 4). Sensitivity analyses showed no heterogeneity with pleiotropy, and “leave-one-out” analyses, funnel plots, and scatter plots further validated the reliability of our results (Figures S25–S36, Supplemental Digital Content, https://links.lww.com/MD/Q196).

### 3.5. Bidirectional causal effects of lung cancer on gut microbiotas and cytokines

Inverse MR analysis showed significant correlations (*P* < .05) between gut microbiota, cytokines, and 3 types of lung cancer. Among them, 19 types of gut microbiota and 6 types of cytokines were significantly associated with LSCC (Tables S9 and S10, Supplemental Digital Content, https://links.lww.com/MD/Q195). Nineteen types of gut microbiota and 1 type of cytokine were significantly associated with LUAD (Table S9 and S10, Supplemental Digital Content, https://links.lww.com/MD/Q195). Four types of gut microbiota and 1 type of cytokine were significantly associated with SCLC (Tables S9 and S10, Supplemental Digital Content, https://links.lww.com/MD/Q195). Sensitivity analyses showed no heterogeneity with pleiotropy. These findings highlight the complex interrelationship between gut microbiota, cytokines, and 3 types of lung cancer, and confirm previous studies that lung cancer patients are often accompanied by disturbances in gut microbiota.^[23–25]^ However, it is important to note that this relationship should be excluded when analyzing the mediating effect of cytokine levels.

### 3.6. Mediation analysis

The results of the mediation analysis showed that cytokines complexly regulate the progression of various types of lung cancer (Table 1). In LSCC, we found that IL-20 could play a mediating role in the abundance of *Faecalicatena lactaris* and CAG-632 in stool, with mediating percentages of 19.11% and 11.00%, respectively. Additionally, IL-24 played a mediating role in the abundance of Atopobiaceae and *Megamonas* in stool, with mediating proportions of 14.85% and 12.79%, respectively. CCL11 played a mediating role in the abundance of Geobacteraceae in stool, with a mediating ratio of 15.68%. CCL8 played a mediating role in the abundance of Veillonellaceae in stool.

**Table 1:** Mediation MR analysis results.

Exposure	Mediator	Outcome	Total effect (β)	A (β)	B (β	Indirect effect (β)	Mediation effect/total effect
*Faecalicatena lactaris* abundance in stool	Interleukin-20 (IL-20) levels	Lung squamous cell carcinoma	−0.3730	0.2160	−0.3300	−0.0713	19.11%
Geobacteraceae abundance in stool	Eotaxin (CCL11) levels	Lung squamous cell carcinoma	−1.6602	−0.7321	0.3555	−0.2603	15.68%
Atopobiaceae abundance in stool	Interleukin-24 (IL-24) levels	Lung squamous cell carcinoma	0.8066	0.3094	0.3871	0.1198	14.85%
*Megamonas* abundance in stool	Interleukin-24 (IL-24) levels	Lung squamous cell carcinoma	−0.2965	−0.0980	0.3871	−0.0379	12.79%
CAG-632 abundance in stool	Interleukin-20 (IL-20) levels	Lung squamous cell carcinoma	−0.4536	0.1512	−0.3300	−0.0499	11.00%
Veillonellaceae abundance in stool	Monocyte chemoattractant protein-1 levels (CCL8)	Lung squamous cell carcinoma	0.3387	0.1181	0.2112	0.0250	7.37%
GCA-900066495 sp900066495 abundance in stool	Transforming growth factor-alpha (TGF-alpha) levels	Lung adenocarcinoma	−0.4707	−0.1625	0.3062	−0.0498	10.57%
Bacillaceae A abundance in stool	Fms-related tyrosine kinase 3 ligand (FIt3L) levels	Lung adenocarcinoma	−2.2562	0.7091	−0.1671	−0.1185	5.25%

Total effect: the causal role of gut microbiota on lung cancer; A: the causal role of gut microbiota on cytokines; B: the causal role of cytokines on lung cancer is independent of the effect of the gut microbiota; β (indirect effect) = β(A) × β(B); the mediated proportion = β (indirect effect)/β (total effect).

MR = Mendelian randomization.

In LUAD, TGF-alpha could play a mediating role in GCA-900066495 sp900066495 abundance in stool with a mediation ratio of 10.57%, respectively. FIt3L also plays a mediating role in the Bacillaceae A abundance in stool with a mediator ratio of 5.25%.

No circulating cytokines were found to mediate the association between gut microbiota and SCLC. Overall, LSCC was more susceptible to the effects of circulating cytokines on the progression of each lung cancer subtype.

## 4. Discussion

Despite some advances in the diagnosis and treatment of lung cancer, it remains the leading cause of cancer-related deaths worldwide.^[26]^ In recent years, the role of gut microbiota in various diseases, including cancer, has received increasing attention. Several studies have suggested that gut microbiota may influence cancer development by modulating host immune responses, inflammatory responses, and other pathways.^[27,28]^ Circulating cytokines, as important components of the immune system, have also been recognized as important biomarkers of lung cancer in recent years. Studies have shown that certain cytokines may increase lung cancer risk by promoting the formation of a tumor microenvironment.^[29]^ However, studies on the causal relationship between gut microbiota and lung cancer are still in the preliminary stage, and it is also unclear whether gut microbiota indirectly affects lung cancer development by influencing circulating cytokines. In view of the above background, the present study combines the latest GWAS data and lung cancer cohort data with the aim of exploring the potential causal relationship between gut microbiota, circulating cytokines, and the 3 types of lung cancer through mediated MR analysis. This will not only help to further understand the pathogenesis of lung cancer, but also provide a scientific basis for future intervention strategies targeting gut microbiota and circulating cytokines.

The findings showed a significant causal association between gut microbiota and 3 types of lung cancer. Specifically, 9 types of gut microbiota, such as *Rhodovulum* abundance in stool, *Clostridium tertium* abundance in stool, and Atopobiaceae abundance in stool, may increase the risk of LSCC. Twelve types of gut microbiota, such as *Azorhizobium* abundance in stool, *Fusobacterium* A abundance in stool, and UBA1191 abundance in stool, may increase the risk of LUAD. Seven types of gut microbiota, such as Poseidoniaceae abundance in stool, Mycobacteriaceae abundance in stool, and *Clostridium* I abundance in stool, may increase the risk of SCLC. Conversely, 10 types of gut microbiota, such as *Coprobacillus* abundance in stool, *Megamonas* abundance in stool, and *Faecalicatena lactaris* abundance in stool, are protective against LSCC. Ten types of gut microbiota, such as *Bacteroides stercoris* abundance in stool, CAG-448 sp003150135 abundance in stool, and CAG-448 abundance in stool, are protective against LUAD. Eight types of gut microbiota, such as CAG-349 abundance in stool, UBA6398 abundance in stool, and *Coprobacillus* abundance in stool, are protective against SCLC. Further analysis of the results revealed that the abundance of *Bifidobacterium longum* and *Coprobacillus* in stool had the same direction of effect on the risk of LSCC and SCLC. Specifically, *Bifidobacterium longum* abundance in stool increased the risk of both types of lung cancer, whereas *Coprobacillus* abundance in stool decreased the disease risk.

*Bifidobacterium longum* is often considered a beneficial intestinal commensal that is able to exert its probiotic function by modulating the host’s immune system.^[30]^ Several studies have shown that *Bifidobacterium longum* has significant anticancer effects, especially in colorectal cancer.^[31,32]^ However, little research has been done on its role in lung cancer. In the present study, however, *Bifidobacterium longum* showed a risk-enhancing effect on 2 types of lung cancer. This suggests that *Bifidobacterium longum* can exhibit different effects in different types of cancers and that its role in lung cancer may be complex. More studies are needed in the future to further reveal the potential mechanism of *Bifidobacterium longum* in lung cancer. *Coprobacillus* is a group of Gram-negative, anaerobic, non-spore-forming bacteria that play an important role in the gut microbiota and can influence host health by regulating intestinal metabolites and immune responses. Although there is a paucity of literature directly examining the relationship between *Coprobacillus* and lung cancer, studies of its role in other cancers provide clues. For example, *Coprobacillus* abundance is significantly reduced in patients with pancreatic cancer, and its reduction may be related to inflammation and immunosuppressive states.^[33]^ Inflammation is also recognized as one of the important drivers of lung cancer, and a study showed that *Coprobacillus* was able to inhibit the growth of inflammation-associated bacteria and reduce the inflammatory response by modulating the host immune response.^[34,35]^ It has also been shown that *Coprobacillus* contributes to the maintenance of gut barrier function through the production of short-chain fatty acids and other metabolites. And the integrity of gut barrier function is critical for preventing inflammation and immune escape in the tumor microenvironment.^[36,37]^ These properties may allow a similar role in lung cancer. Our findings enhance the understanding of the complex interactions between gut microbiota and lung cancer.

Similar to gut microbiota, circulating cytokines play an important role in the development and progression of all 3 types of lung cancer. Specifically, 4 cytokines, IL-24, CCL11, EN-RAGE, and CCL8, may increase the risk of LSCC, 2 cytokines, TGF-alpha and IL-10, may increase the risk of LUAD, and FGF-23 may increase the disease risk of SCLC. On the contrary, 2 cytokines, TRAIL and IL-20, may decrease the risk of LSCC. Two cytokines, TRAIL and FIt3L, may decrease the risk of LUAD. Two cytokines, IL-15RA and CASP-8, may reduce the risk of SCLC. An interesting phenomenon that can be found by analyzing the results is that the TRAIL can play a role in reducing the risk for both LSCC and LUAD. TRAIL is an apoptosis-inducing factor mediated by the TNF superfamily, and by binding to its receptors DR4 and DR5, it selectively induces apoptosis in cancer cells without significant toxicity to normal cells.^[38]^ Although the mechanism of TRAIL in lung cancer is not completely clear, studies have revealed its potential pathways of action. In LSCC, it has been shown that TRAIL is able to inhibit tumor cell proliferation and induce apoptosis by activating the death receptor-mediated apoptotic pathway.^[39]^ In addition, the role of TRAIL in LUAD has also gained attention, and it was found that TRAIL was able to enhance apoptosis susceptibility of tumor cells by up-regulating specific signaling pathways, such as ERK and PI3K/Akt/Bad pathways.^[40,41]^ This is consistent with our findings that TRAIL has a risk-reducing effect on both squamous cell carcinoma and adenocarcinoma of the lung, which provides a new idea for the application of TRAIL in lung cancer therapy. It is worth noting that the OR values for the impact of some cytokines on lung cancer risk are relatively small. Although these values may appear minor, for widespread and continuous exposure, the population attributable risk may become significant. This is particularly true in a multifactorial disease like lung cancer, where the effect of a single factor is inherently limited. The value lies in revealing intervenable biological pathways, providing new targets for combined intervention or risk stratification.

We performed MR analysis of selected gut microbiota and circulating cytokines in the above 3 types of lung cancer to further explore the potential mediating role of gut microbiota exposure in these important mediators. The results showed that the same gut microbiota could significantly associate with multiple cytokines. For example, GCA-900066495 sp900066495 abundance in stool was significantly negatively correlated with both TRAIL and TGF-alpha. In addition, lung cancer may also affect gut microbiota and cytokines. Therefore, we performed a reverse MR analysis to investigate whether there was an inverse causal relationship between various types of lung cancer, gut microbiota and cytokines. The results showed significant correlations (*P* < .05) between gut microbiota, cytokines, and 3 types of lung cancer. Among them, 19 gut microbiota and 6 cytokines were significantly associated with LSCC. Nineteen gut microbiota and 1 cytokine were significantly associated with LUAD. Four gut microbiota and 1 cytokine were significantly associated with SCLC. These findings highlight the complex interrelationship between gut microbiota, cytokines, and 3 types of lung cancer, and confirm previous studies showing that lung cancer patients often experience disturbances in gut microbiota.^[23–25]^ However, it is important to note that this relationship should be excluded when analyzing the mediating effect of cytokines.

The results of our mediation analyses suggest that cytokines complexly regulate the progression of various types of lung cancer. In LSCC, we found that IL-20 could play a mediating role in *Faecalicatena lactaris* abundance in stool, and CAG-632 abundance in stool, with mediating proportions of 19.11% and 11.00%, respectively. In addition, IL-24 played a mediator role in Atopobiaceae abundance in stool and *Megamonas* abundance in stool with mediator proportions of 14.85% and 12.79%, respectively. CCL11 played a mediating role in the abundance of Geobacteraceae in stool, with a mediating ratio of 15.68%. CCL8 played a mediating role in the abundance of Veillonellaceae in stool. In LUAD, TGF-alpha could play a mediating role in GCA-900066495 sp900066495 abundance in stool with a mediation ratio of 10.57%, respectively. No circulating cytokines were found to mediate the association between gut microbiota and SCLC. Overall, LSCC was more susceptible to circulating cytokine in the influence of gut microbiota on the progression in each lung cancer subtype. These findings provide direction for intervention strategies based on gut microbiota and circulating cytokines.

Although this study provides strong evidence to support the role of gut microbiota and circulating cytokines in lung cancer, several limitations remain. First, although multiple statistical methods were used to enhance the robustness of the results, these methods do not completely exclude all potential biases. Second, this study was based on data from the European population only, and caution should be exercised when generalizing these findings to populations of different ethnic backgrounds. Future studies could further explore the specific molecular mechanisms of microbiota and circulating cytokines in lung cancer. For example, in-depth analysis of how gut microbiota affects circulating cytokine levels through metabolites or immune signaling pathways through transcriptomics and metabolomics techniques. In addition, combined with multivariate MR analysis, the combined effects of multiple exposure factors on lung cancer risk can be assessed simultaneously.

## 5. Conclusion

In this study, based on the latest GWAS data, we comprehensively revealed the causal relationship between 473 types of gut microbiota, 91 types of circulating cytokines, and 3 types of lung cancer by mediator MR analysis. Through mediated analysis, we identified 6 important circulating cytokines (IL-20, CCL11, IL-24, CCL8, TGF-alpha, and FIt3L), which intricately regulate the onset and progression of the 3 types of lung cancer. LSCC is more susceptible to circulating cytokines than LUAD and SCLC. This finding not only enriches the etiological study of lung cancer, but also provides a theoretical basis for future intervention strategies based on gut microbiota and circulating cytokines.

## Author contributions

**Conceptualization:** Jianjie Zhang, Chengyi Liu, Changzhen Zhang, Xin Liu.

**Data curation:** Wei Shen, Ruolin Hou, Xin Liu.

**Formal analysis:** Wei Shen, Ruolin Hou, Chengyi Liu, Xin Liu.

**Funding acquisition:** Wei Shen, Ruolin Hou, Xin Liu.

**Investigation:** Xin Liu.

**Methodology:** Ruolin Hou, Jianjie Zhang, Chengyi Liu, Changzhen Zhang, Xin Liu.

**Project administration:** Wei Shen, Ruolin Hou, Xin Liu.

**Supervision:** Jianjie Zhang, Xin Liu.

**Visualization:** Wei Shen, Xin Liu.

**Writing – original draft:** Wei Shen, Ruolin Hou, Xin Liu.

**Writing – review & editing:** Wei Shen, Ruolin Hou, Jianjie Zhang, Chengyi Liu, Changzhen Zhang, Xin Liu.

## Supplementary Material
