# Supplementary material for: Gut microbiota influences lung cancer risk through circulating cytokines—Insights from a Mendelian randomization study
Source: Medicine (Baltimore). 2025 Oct 3;104(40):e44897. doi: 10.1097/MD.0000000000044897 (PMC12499803; doi:10.1097/MD.0000000000044897)
Supplement: Supplementary file 2 [file medi-104-e44897-s002.pdf]

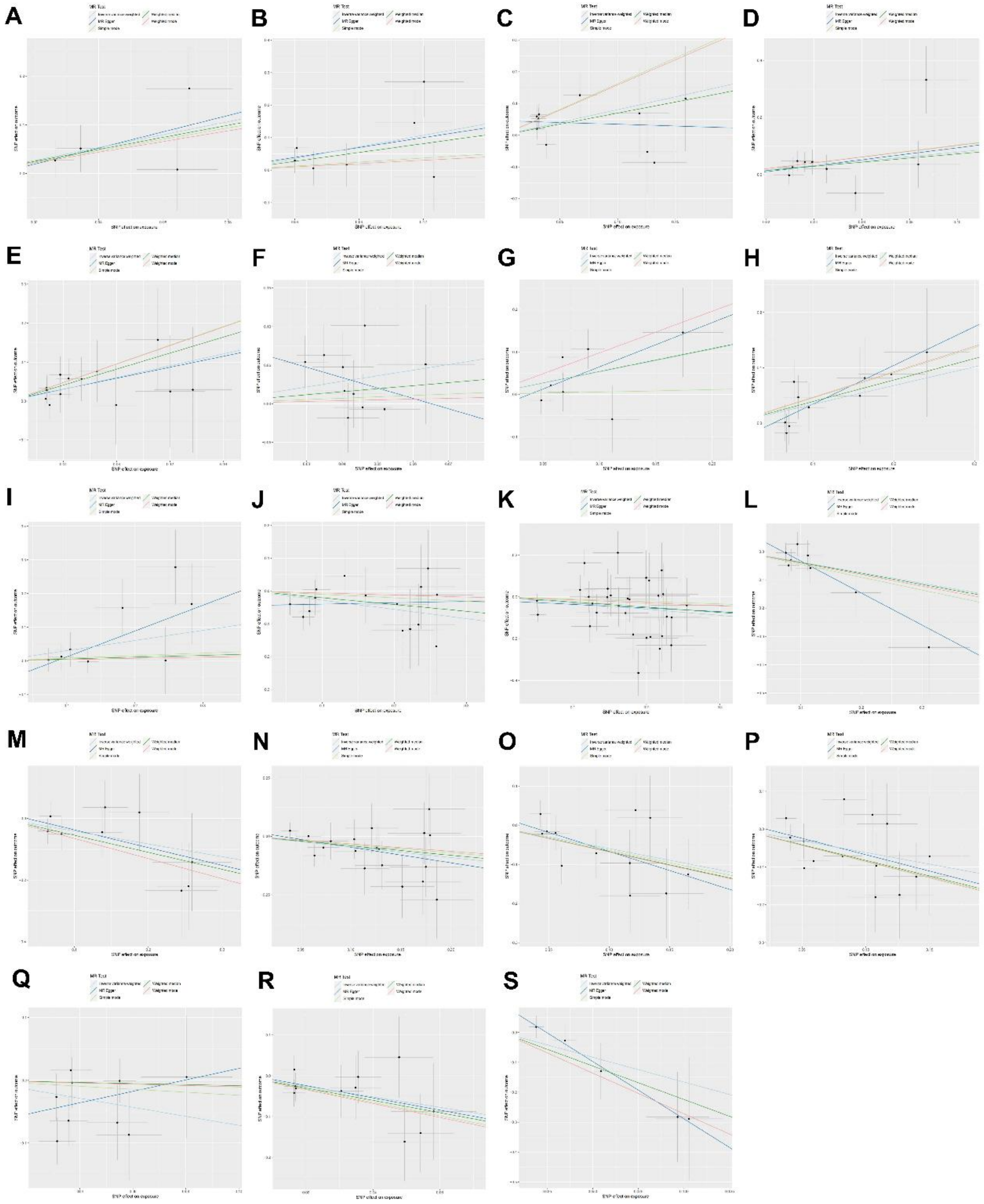

Supplementary figure 1. Scatter plot of MR analysis for 19 gut microbiota on lung squamous cell carcinoma (A-S). (A) Rhodovulum abundance in stool; (B) Clostridium tertium abundance in stool; (C) Atopobiaceae abundance in stool; (D) Methanobrevibacter B abundance in stool; (E) Dysgonomonadaceae abundance in stool; (F) Gillisia abundance in stool; (G) CAG-822 sp000432855 abundance in stool; (H) Veillonellaceae abundance in stool; (I) Bifidobacterium longum abundance in stool; (J) Coprobacillus abundance in stool; (K) Megamonas abundance in stool; (L) Faecalicatena lactaris abundance in stool; (M) CAG-485 sp002362485 abundance in stool; (N) CAG-632 abundance in stool; (O) GCA-900066755 abundance in stool; (P) GCA-900066755 sp900066755 abundance in stool; (Q) UBA3855 sp900316885 abundance in stool; (R) SAR324 abundance in stool; (S) Geobacteraceae abundance in stool.

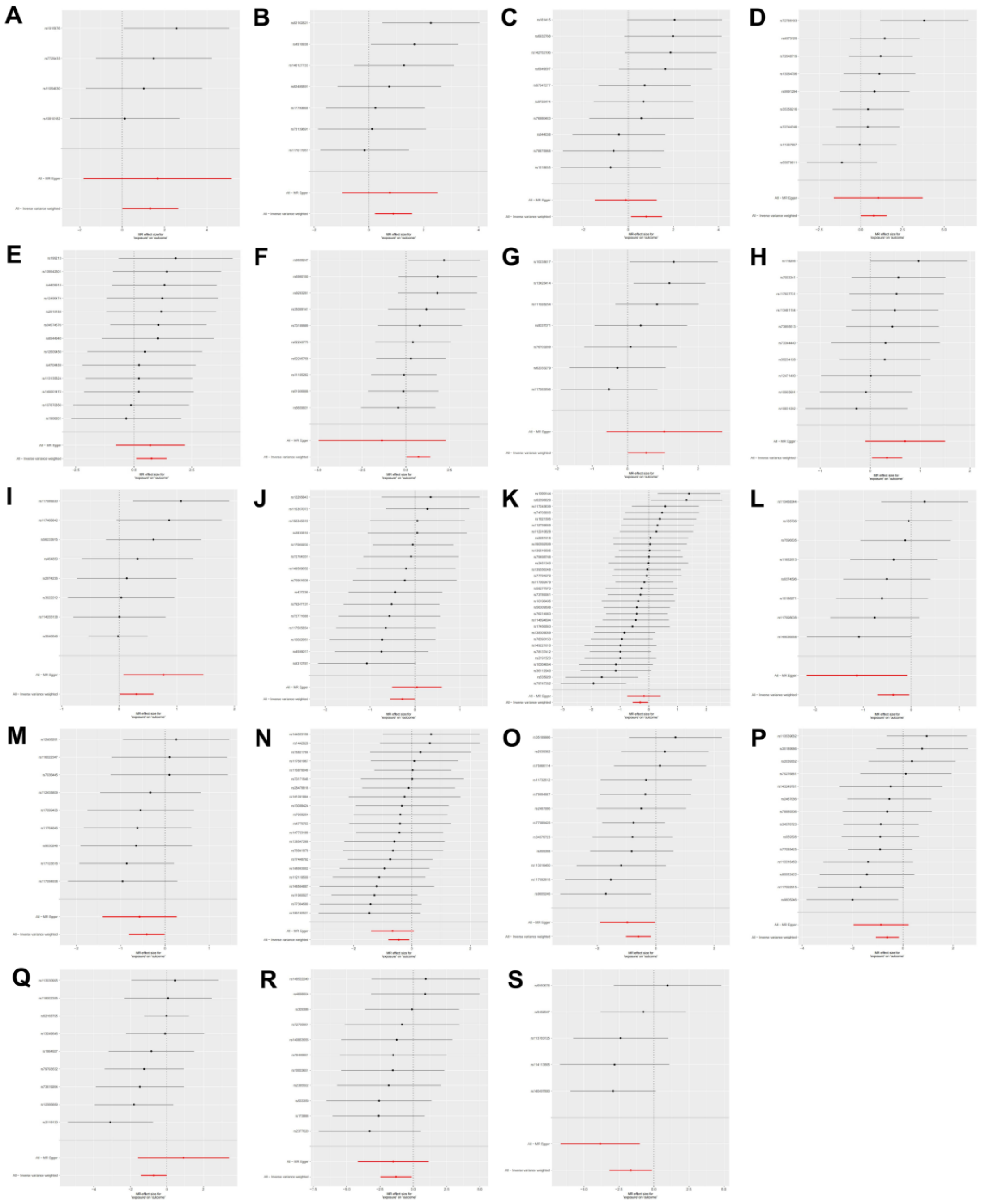

Supplementary figure 2. Forest plot of MR analysis results for 19 gut microbiota on lung squamous cell carcinoma (A-S). (A) Rhodovulum abundance in stool; (B) Clostridium tertium abundance in stool; (C) Atopobiaceae abundance in stool; (D) Methanobrevibacter B abundance in stool; (E) Dysgonomonadaceae abundance in stool; (F) Gillisia abundance in stool; (G) CAG-822 sp000432855 abundance in stool; (H) Veillonellaceae abundance in stool; (I) Bifidobacterium longum abundance in stool; (J) Coprobacillus abundance in stool; (K) Megamonas abundance in stool; (L) Faecalicatena lactaris abundance in stool; (M) CAG-485 sp002362485 abundance in stool; (N) CAG-632 abundance in stool; (O) GCA-900066755 abundance in stool; (P) GCA-900066755 sp900066755 abundance in stool; (Q) UBA3855 sp900316885 abundance in stool; (R) SAR324 abundance in stool; (S) Geobacteraceae abundance in stool.

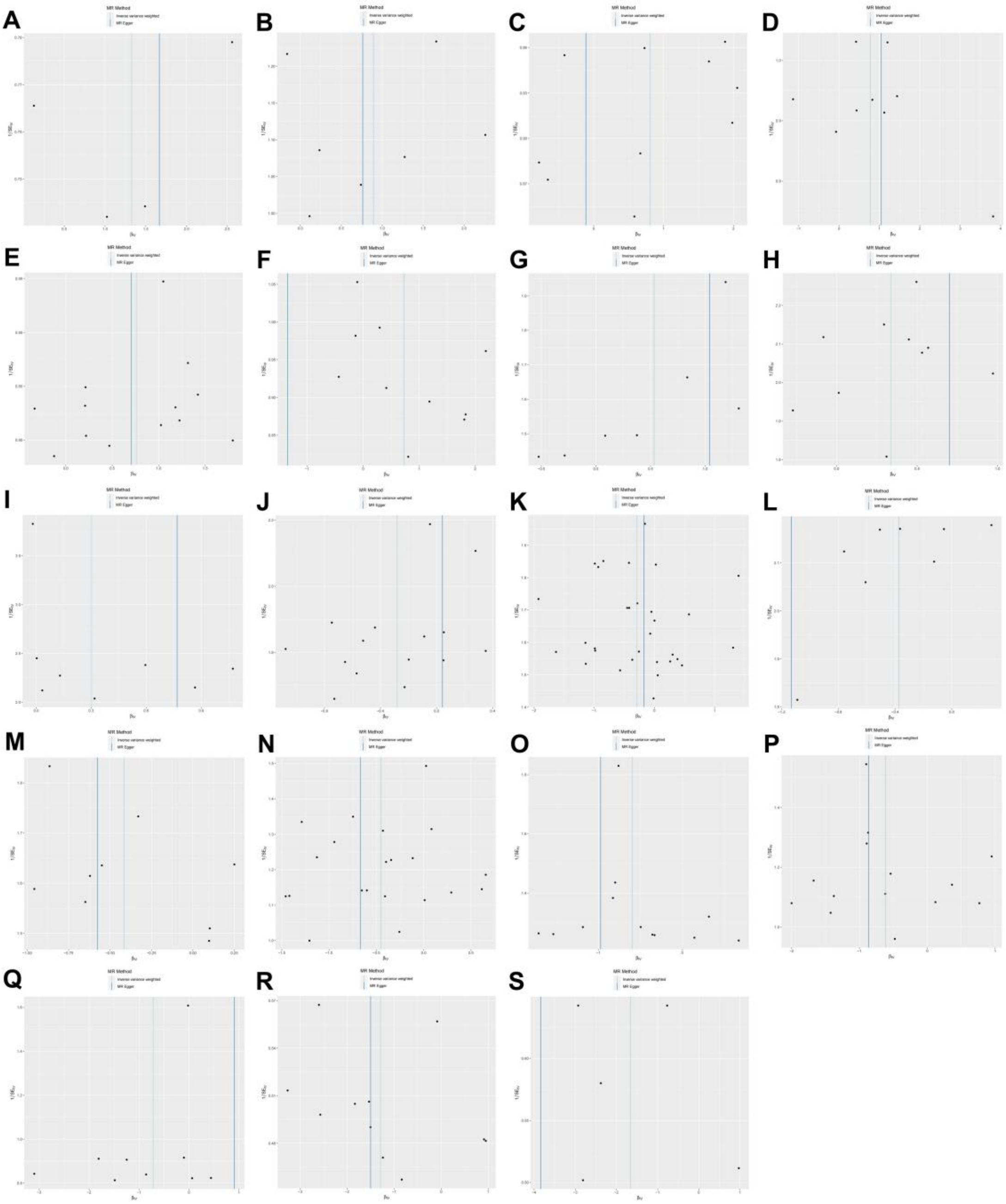

Supplementary figure 3. Funnel plot of MR analysis for 19 gut microbiota on lung squamous cell carcinoma (A-S). (A) Rhodovulum abundance in stool; (B) Clostridium tertium abundance in stool; (C) Atopobiaceae abundance in stool; (D) Methanobrevibacter B abundance in stool; (E) Dysgonomonadaceae abundance in stool; (F) Gillisia abundance in stool; (G) CAG-822 sp000432855 abundance in stool; (H) Veillonellaceae abundance in stool; (I) Bifidobacterium longum abundance in stool; (J) Coprobacillus abundance in stool; (K) Megamonas abundance in stool; (L) Faecalicatena lactaris abundance in stool; (M) CAG-485 sp002362485 abundance in stool; (N) CAG-632 abundance in stool; (O) GCA-900066755 abundance in stool; (P) GCA-900066755 sp900066755 abundance in stool; (Q) UBA3855 sp900316885 abundance in stool; (R) SAR324 abundance in stool; (S) Geobacteraceae abundance in stool.

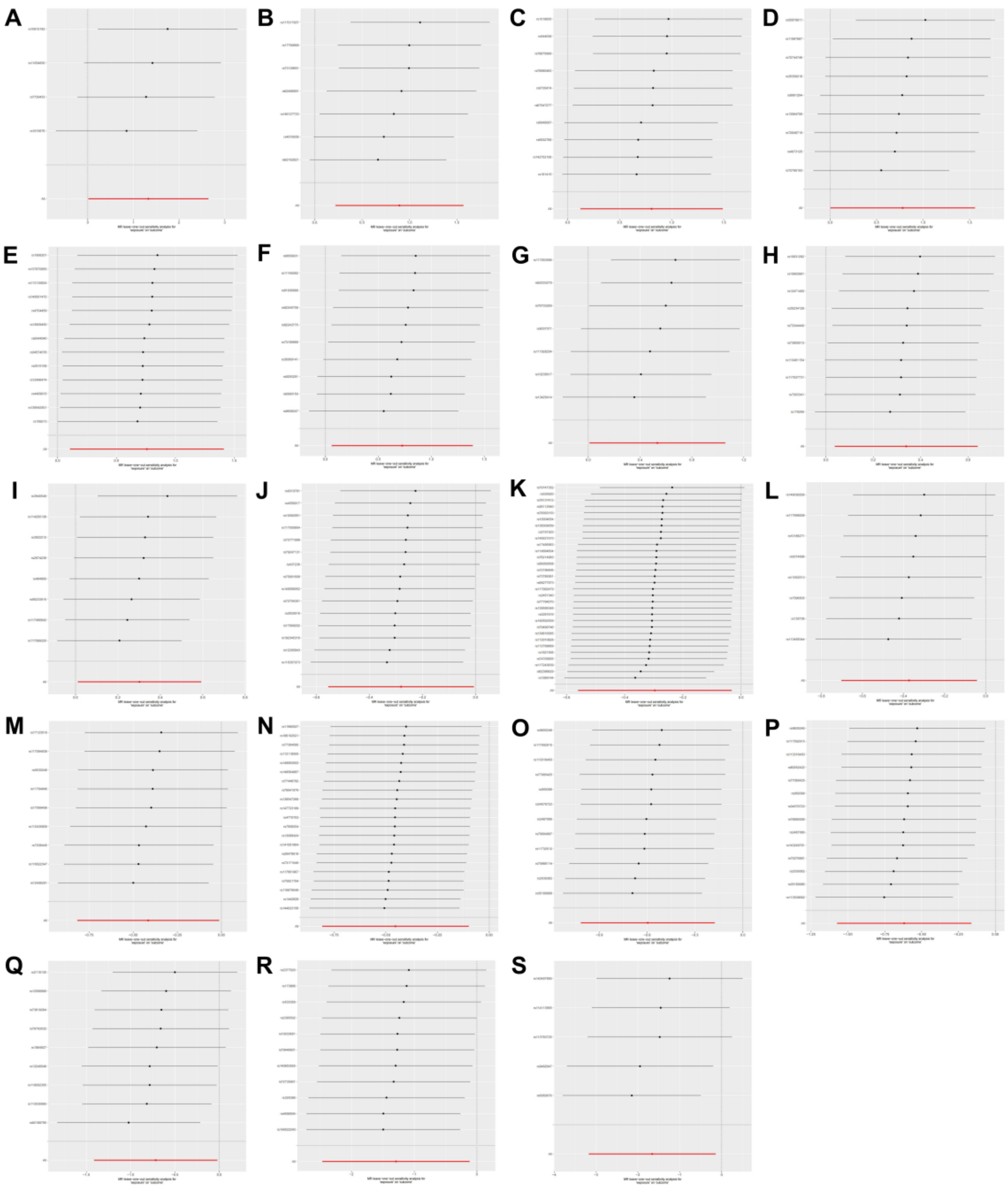

Supplementary figure 4. Leave-one-out plot of MR analysis for 19 gut microbiota on lung squamous cell carcinoma (A-S). (A) Rhodovulum abundance in stool; (B) Clostridium tertium abundance in stool; (C) Atopobiaceae abundance in stool; (D) Methanobrevibacter B abundance in stool; (E) Dysgonomonadaceae abundance in stool; (F) Gillisia abundance in stool; (G) CAG-822 sp000432855 abundance in stool; (H) Veillonellaceae abundance in stool; (I) Bifidobacterium longum abundance in stool; (J) Coprobacillus abundance in stool; (K) Megamonas abundance in stool; (L) Faecalicatena lactaris abundance in stool; (M) CAG-485 sp002362485 abundance in stool; (N) CAG-632 abundance in stool; (O) GCA-900066755 abundance in stool; (P) GCA-900066755 sp900066755 abundance in stool; (Q) UBA3855 sp900316885 abundance in stool; (R) SAR324 abundance in stool; (S) Geobacteraceae abundance in stool.

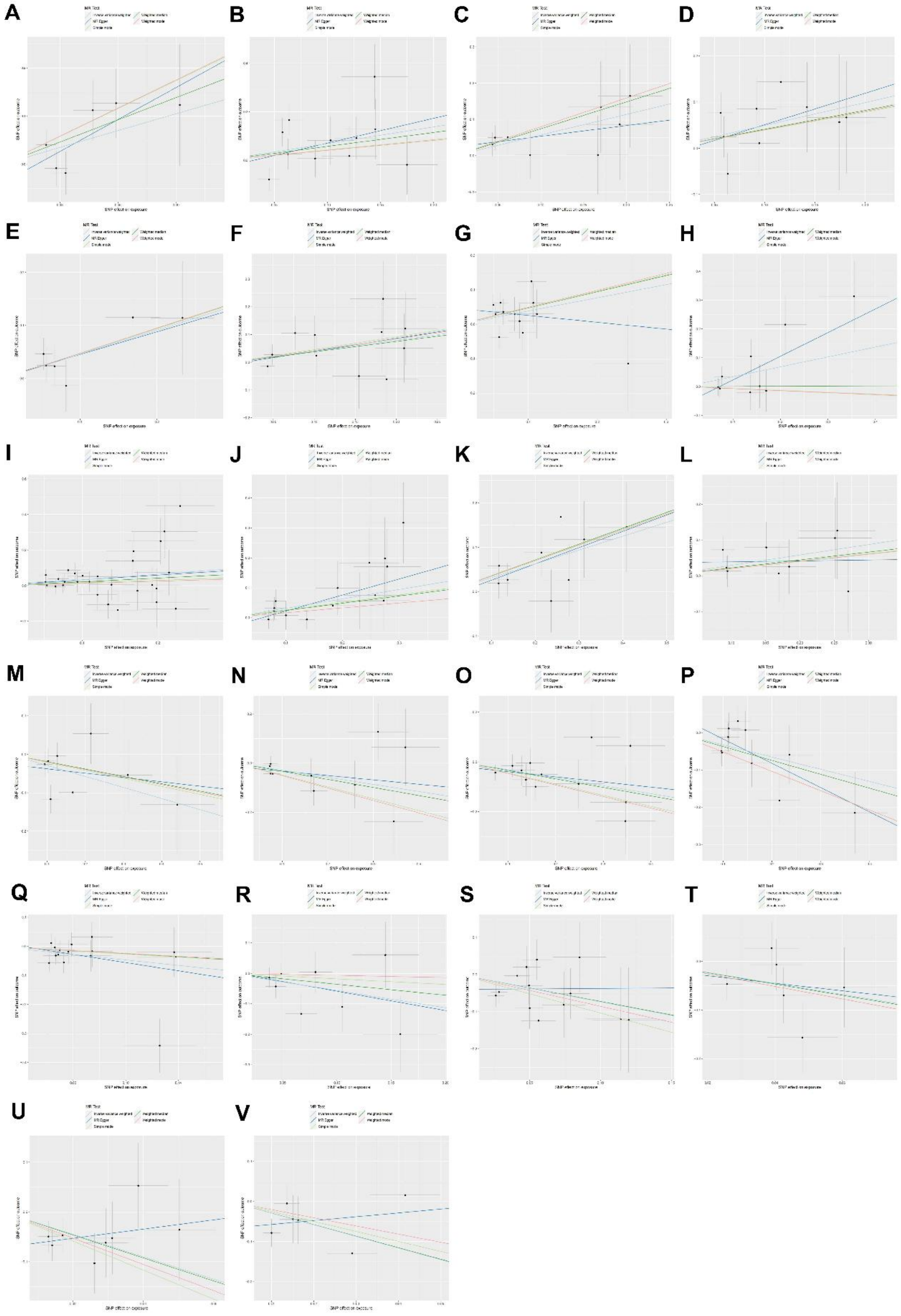

Supplementary figure 5. Scatter plot of MR analysis for 22 gut microbiota on lung adenocarcinoma (A-V). (A) Azorhizobium abundance in stool; (B) Fusobacterium A abundance in stool; (C) UBA1191 abundance in stool; (D) CAG-485 sp002404675 abundance in stool; (E) Coprobacter secundus abundance in stool; (F) Acidaminococcus fermentans abundance in stool; (G) Enteroscipio abundance in stool; (H) CAG-177 sp003538135 abundance in stool; (I) Succinivibrio abundance in stool; (J) Bifidobacterium angulatum abundance in stool; (K) Bacteroides eggerthii abundance in stool; (L) Turicibacter abundance in stool; (M) Bacteroides stercoris abundance in stool; (N) CAG-448 sp003150135 abundance in stool; (O) CAG-448 abundance in stool; (P) Victivallis sp002998355 abundance in stool; (Q) GCA-900066495 sp900066495 abundance in stool; (R) Clostridium M sp001304855 abundance in stool; (S) Providencia abundance in stool; (T) Magnetospirillum A abundance in stool; (U) Bacillaceae A abundance in stool; (V) Paenibacillales abundance in stool.

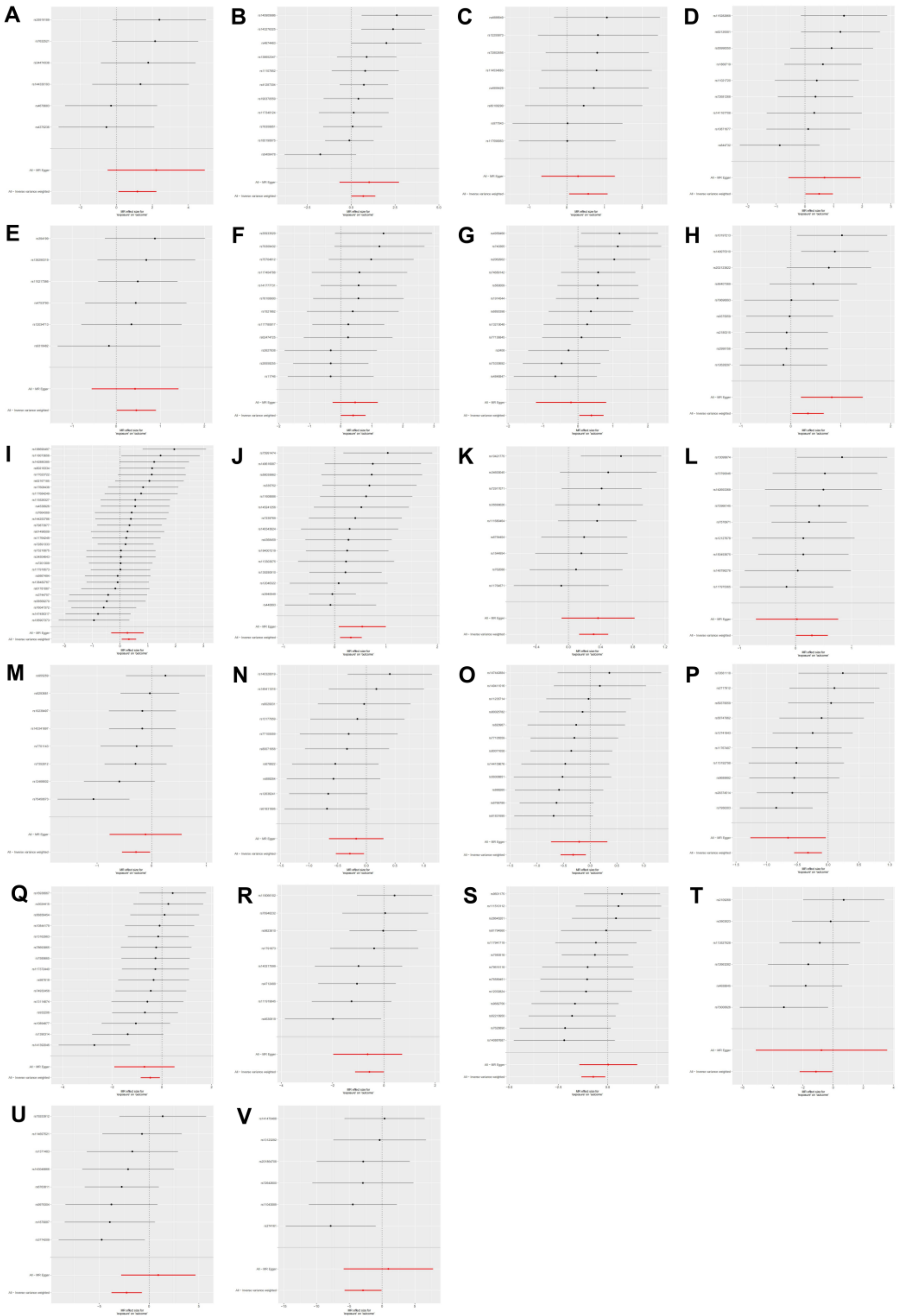

Supplementary figure 6. Forest plot of MR analysis results for 22 gut microbiota on lung adenocarcinoma (A-V). (A) Azorhizobium abundance in stool; (B) Fusobacterium A abundance in stool; (C) UBA1191 abundance in stool; (D) CAG-485 sp002404675 abundance in stool; (E) Coprobacter secundus abundance in stool; (F) Acidaminococcus fermentans abundance in stool; (G) Enteroscipio abundance in stool; (H) CAG-177 sp003538135 abundance in stool; (I) Succinivibrio abundance in stool; (J) Bifidobacterium angulatum abundance in stool; (K) Bacteroides eggerthii abundance in stool; (L) Turicibacter abundance in stool; (M) Bacteroides stercoris abundance in stool; (N) CAG-448 sp003150135 abundance in stool; (O) CAG-448 abundance in stool; (P) Victivallis sp002998355 abundance in stool; (Q) GCA-900066495 sp900066495 abundance in stool; (R) Clostridium M sp001304855 abundance in stool; (S) Providencia abundance in stool; (T) Magnetospirillum A abundance in stool; (U) Bacillaceae A abundance in stool; (V) Paenibacillales abundance in stool.

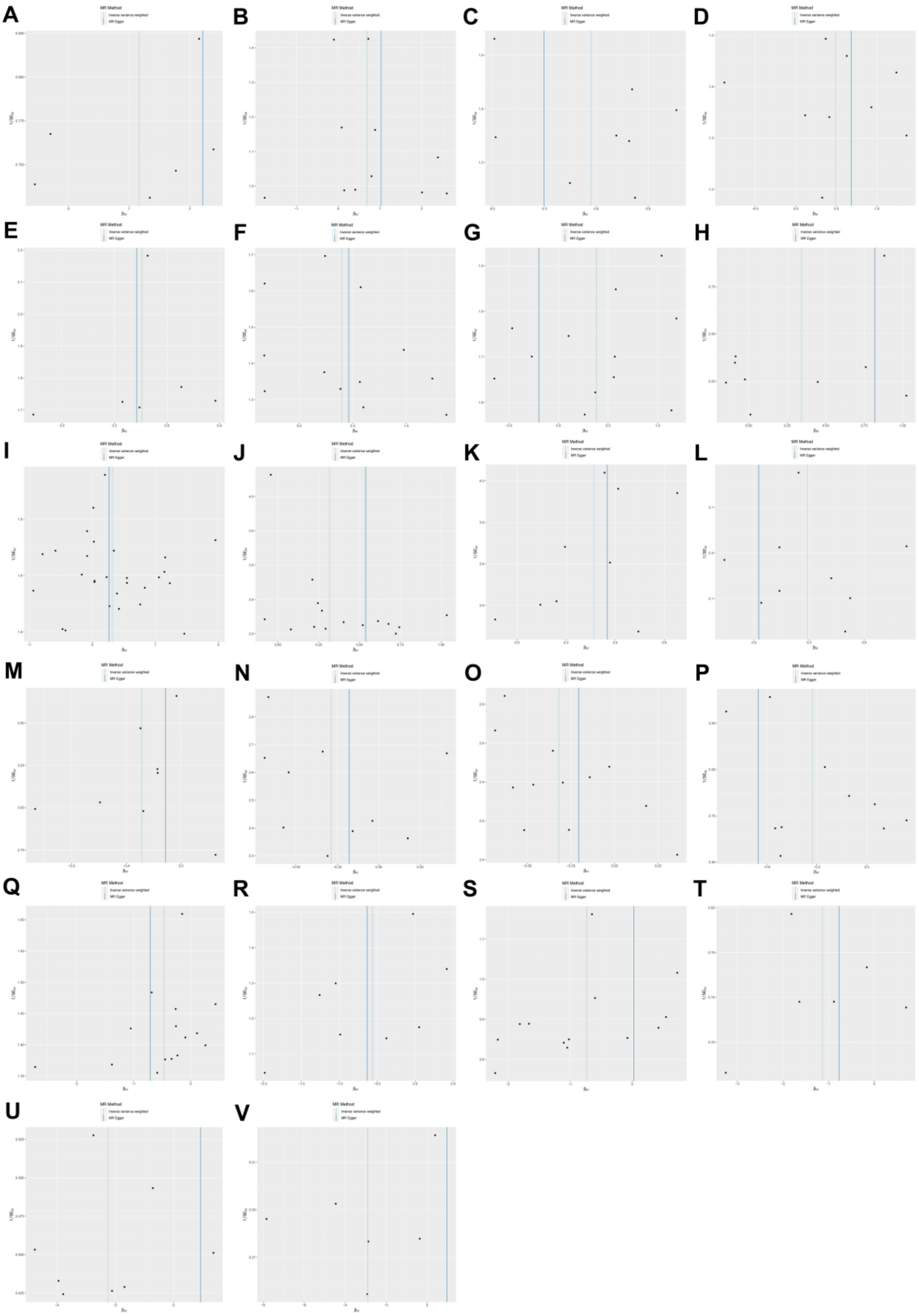

Supplementary figure 7. Funnel plot of MR analysis for 22 gut microbiota on lung adenocarcinoma (A-V). (A) Azorhizobium abundance in stool; (B) Fusobacterium A abundance in stool; (C) UBA1191 abundance in stool; (D) CAG-485 sp002404675 abundance in stool; (E) Coprobacter secundus abundance in stool; (F) Acidaminococcus fermentans abundance in stool; (G) Enteroscipio abundance in stool; (H) CAG-177 sp003538135 abundance in stool; (I) Succinivibrio abundance in stool; (J) Bifidobacterium angulatum abundance in stool; (K) Bacteroides eggerthii abundance in stool; (L) Turicibacter abundance in stool; (M) Bacteroides stercoris abundance in stool; (N) CAG-448 sp003150135 abundance in stool; (O) CAG-448 abundance in stool; (P) Victivallis sp002998355 abundance in stool; (Q) GCA-900066495 sp900066495 abundance in stool; (R) Clostridium M sp001304855 abundance in stool; (S) Providencia abundance in stool; (T) Magnetospirillum A abundance in stool; (U) Bacillaceae A abundance in stool; (V) Paenibacillales abundance in stool.

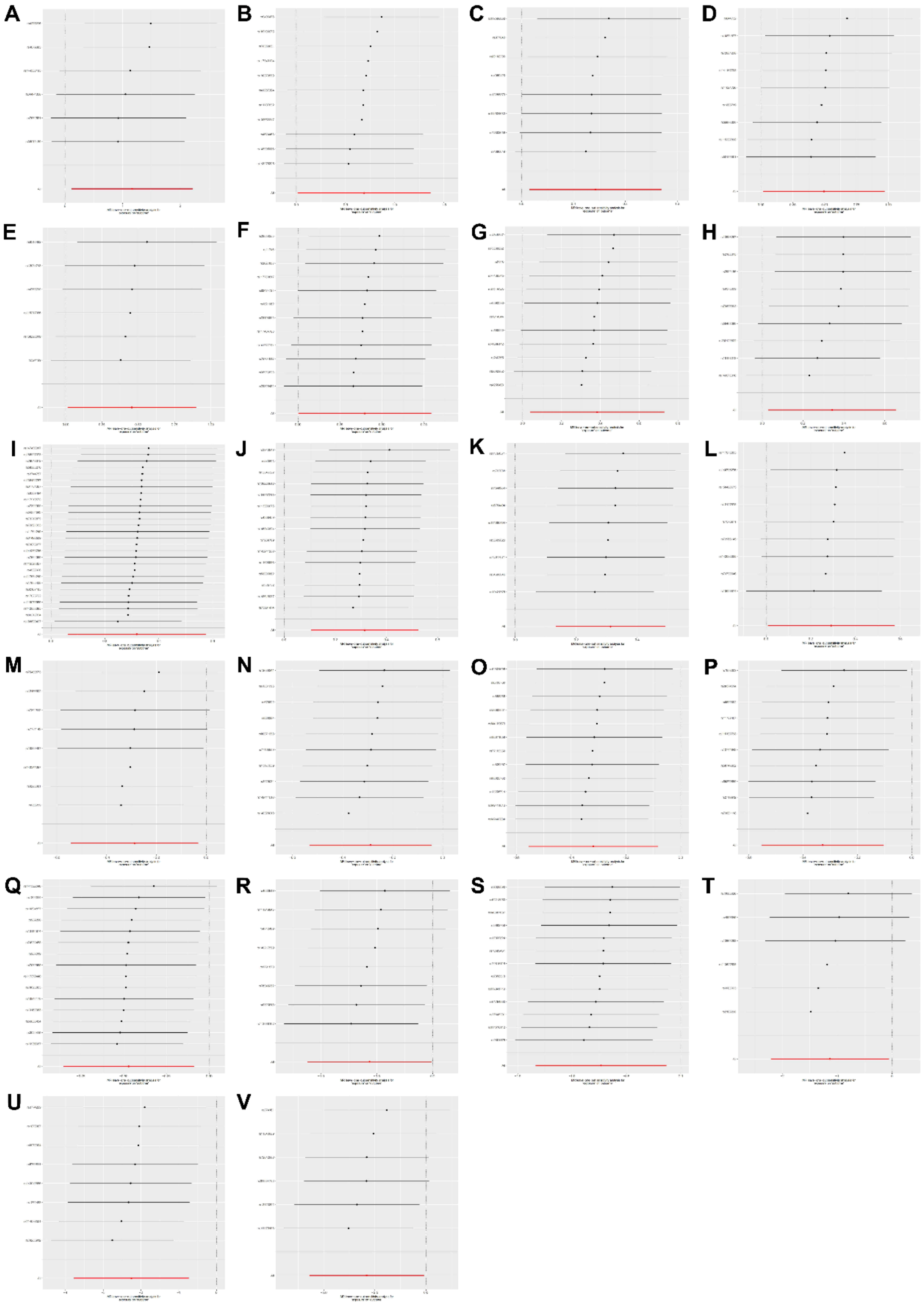

Supplementary figure 8. Leave-one-out plot of MR analysis for 22 gut microbiota on lung adenocarcinoma (A-V). (A) Azorhizobium abundance in stool; (B) Fusobacterium A abundance in stool; (C) UBA1191 abundance in stool; (D) CAG-485 sp002404675 abundance in stool; (E) Coprobacter secundus abundance in stool; (F) Acidaminococcus fermentans abundance in stool; (G) Enteroscipio abundance in stool; (H) CAG-177 sp003538135 abundance in stool; (I) Succinivibrio abundance in stool; (J) Bifidobacterium angulatum abundance in stool; (K) Bacteroides eggerthii abundance in stool; (L) Turicibacter abundance in stool; (M) Bacteroides stercoris abundance in stool; (N) CAG-448 sp003150135 abundance in stool; (O) CAG-448 abundance in stool; (P) Victivallis sp002998355 abundance in stool; (Q) GCA-900066495 sp900066495 abundance in stool; (R) Clostridium M sp001304855 abundance in stool; (S) Providencia abundance in stool; (T) Magnetospirillum A abundance in stool; (U) Bacillaceae A abundance in stool; (V) Paenibacillales abundance in stool.

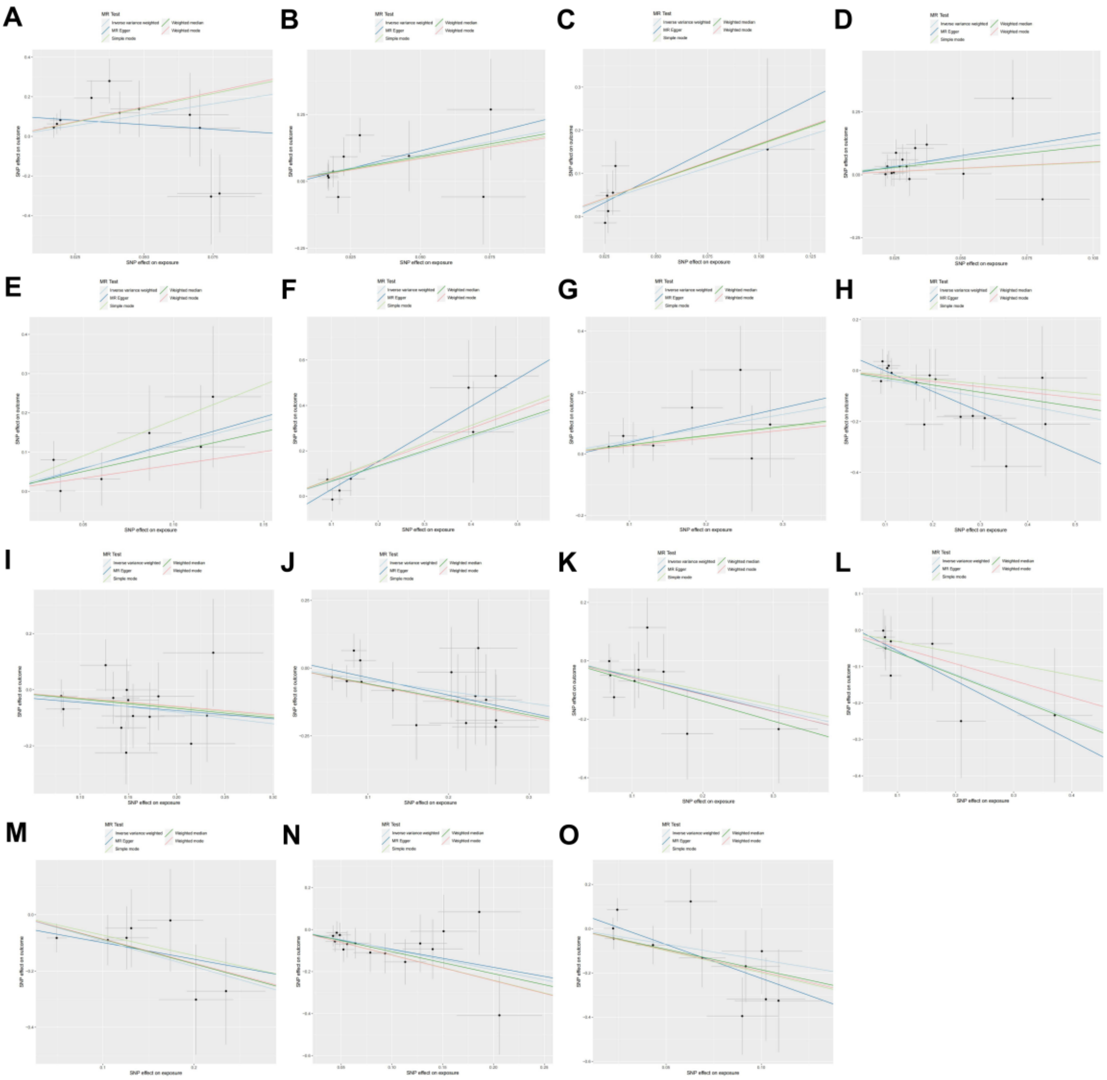

Supplementary figure 9. Scatter plot of MR analysis for 15 gut microbiota on small cell lung cancer (A-O). (A) Poseidoniaceae abundance in stool; (B) Mycobacteriaceae abundance in stool; (C) Clostridium I abundance in stool; (D) SM23-33 abundance in stool; (E) Dorea abundance in stool; (F) Bacteroides A abundance in stool; (G) Bifidobacterium longum abundance in stool; (H) CAG-349 abundance in stool; (I) UBA6398 abundance in stool; (J) Coprobacillus abundance in stool; (K) KLE1615 sp900066985 abundance in stool; (L) KLE1615 abundance in stool; (M) CAG-145 abundance in stool; (N) UBA737 sp002451855 abundance in stool; (O) Stappia abundance in stool.

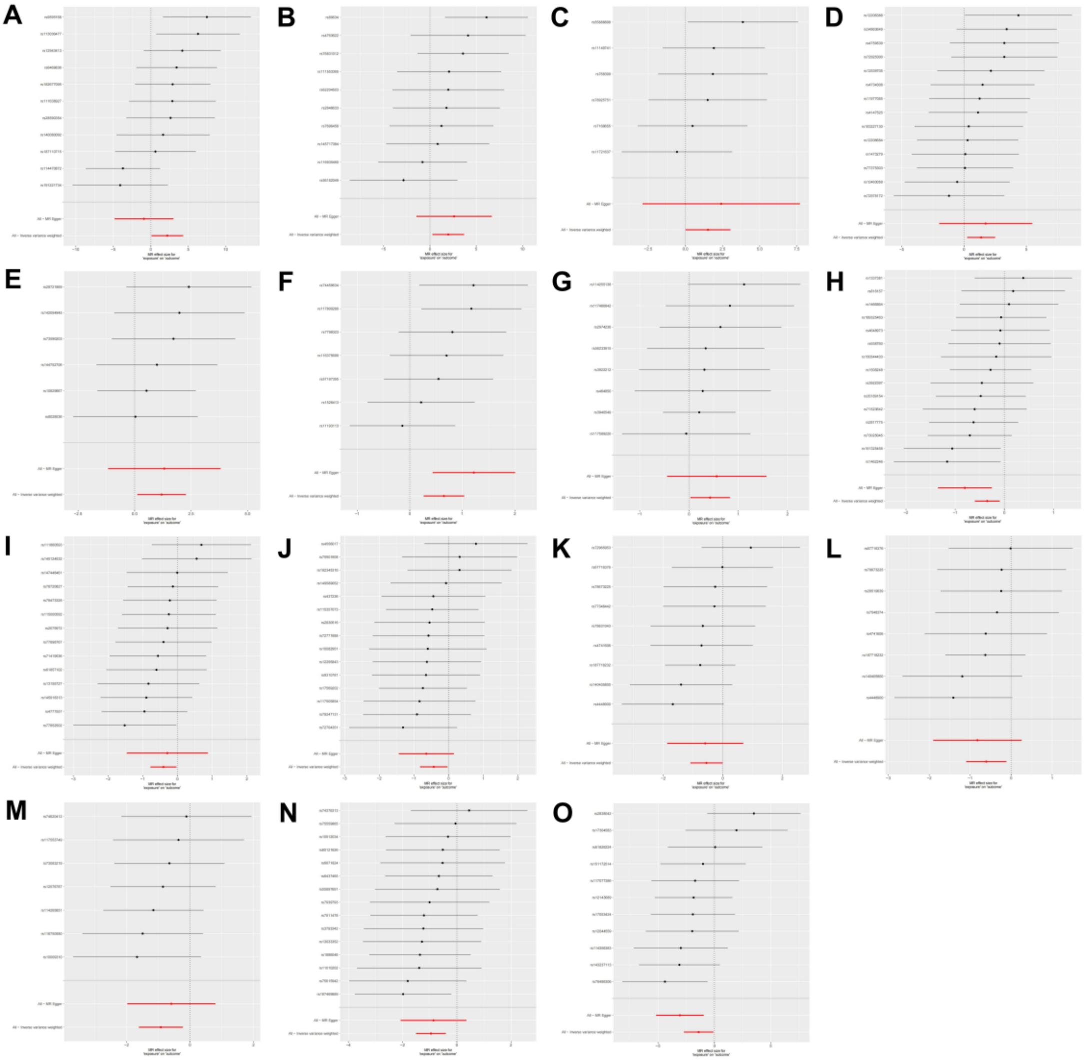

Supplementary figure 10. Forest plot of MR analysis results for 15 gut microbiota on small cell lung cancer (A-O). (A) Poseidoniaceae abundance in stool; (B) Mycobacteriaceae abundance in stool; (C) Clostridium I abundance in stool; (D) SM23-33 abundance in stool; (E) Dorea abundance in stool; (F) Bacteroides A abundance in stool; (G) Bifidobacterium longum abundance in stool; (H) CAG-349 abundance in stool; (I) UBA6398 abundance in stool; (J) Coprobacillus abundance in stool; (K) KLE1615 sp900066985 abundance in stool; (L) KLE1615 abundance in stool; (M) CAG-145 abundance in stool; (N) UBA737 sp002451855 abundance in stool; (O) Stappia abundance in stool.

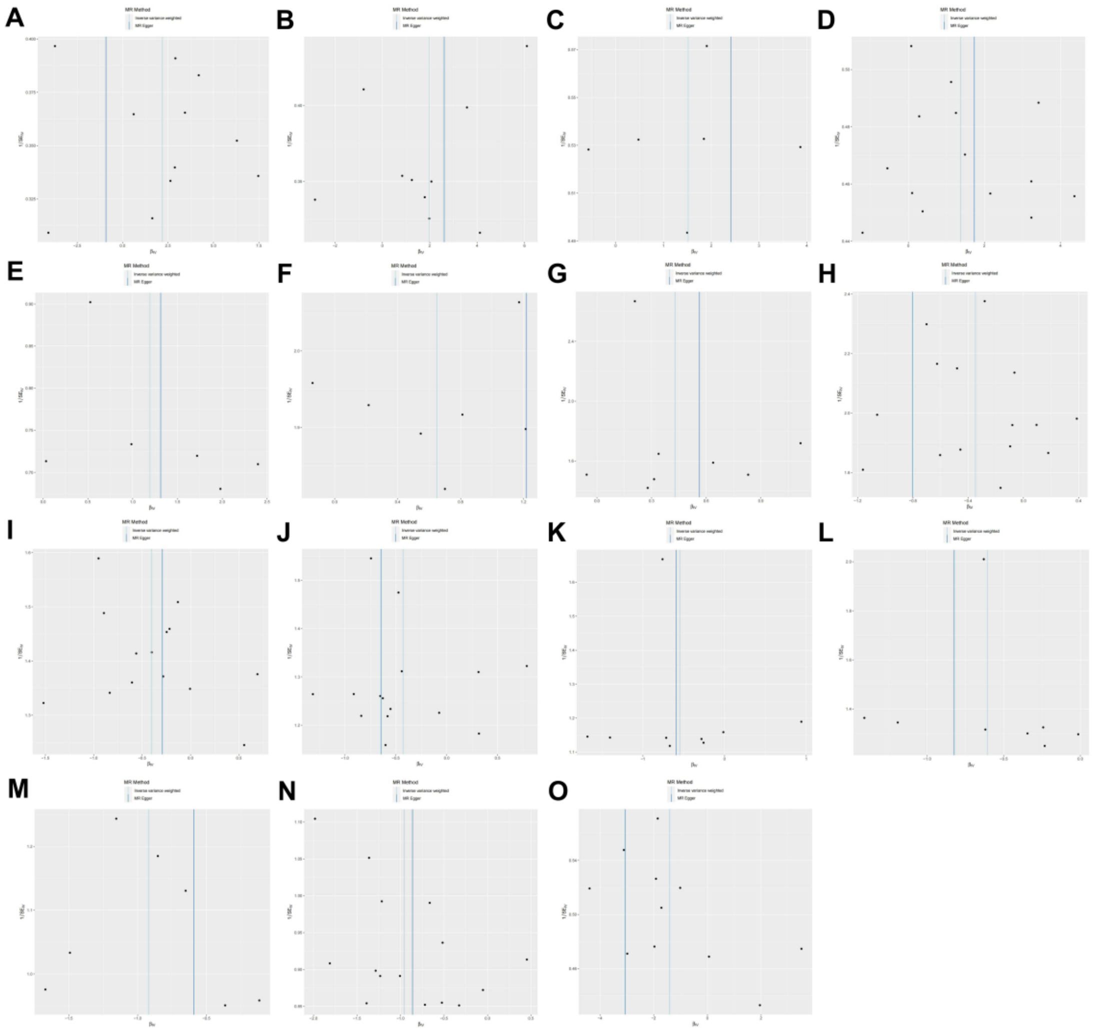

Supplementary figure 11. Funnel plot of MR analysis for 15 gut microbiota on small cell lung cancer (A-O). (A) Poseidoniaceae abundance in stool; (B) Mycobacteriaceae abundance in stool; (C) Clostridium I abundance in stool; (D) SM23-33 abundance in stool; (E) Dorea abundance in stool; (F) Bacteroides A abundance in stool; (G) Bifidobacterium longum abundance in stool; (H) CAG-349 abundance in stool; (I) UBA6398 abundance in stool; (J) Coprobacillus abundance in stool; (K) KLE1615 sp900066985 abundance in stool; (L) KLE1615 abundance in stool; (M) CAG-145 abundance in stool; (N) UBA737 sp002451855 abundance in stool; (O) Stappia abundance in stool.

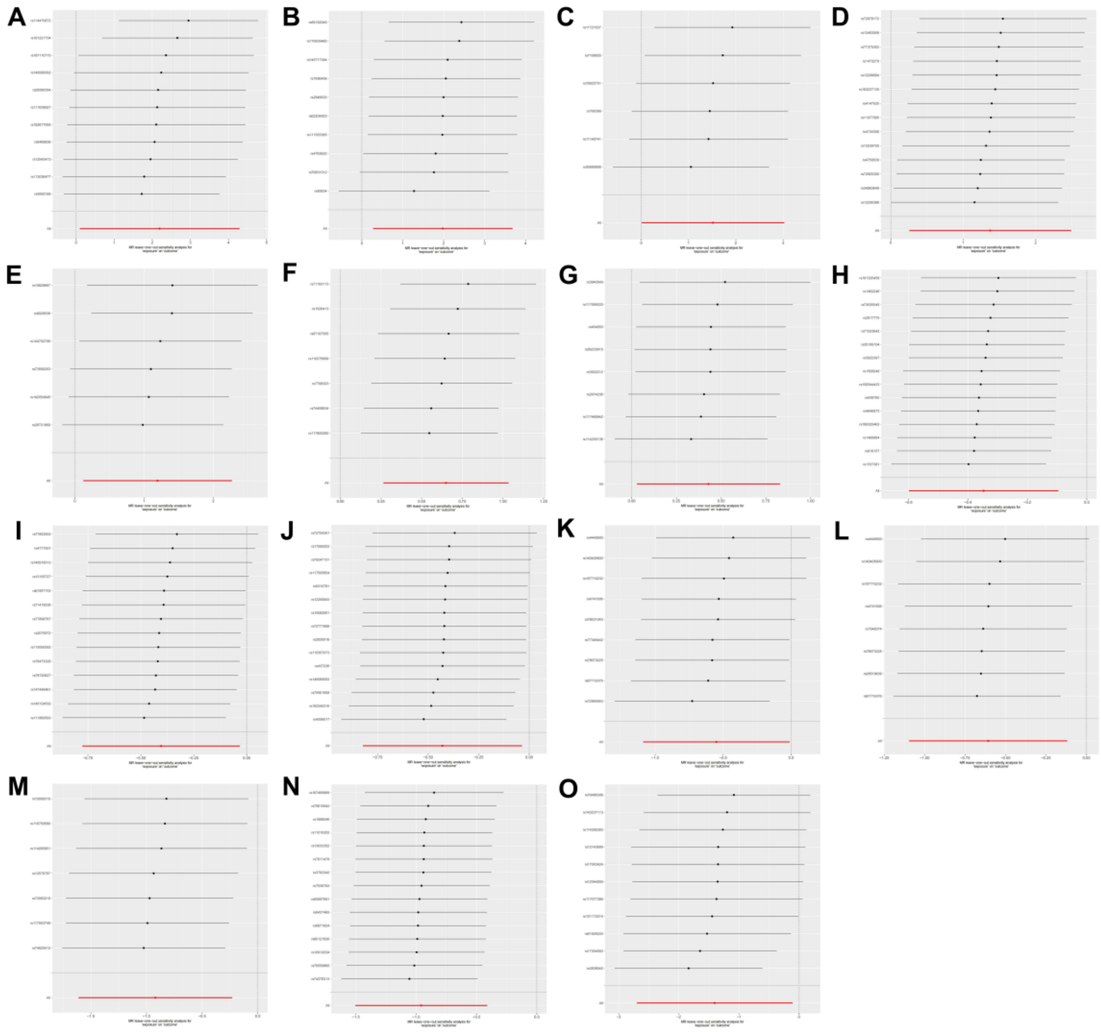

Supplementary figure 12. Leave-one-out plot of MR analysis for 15 gut microbiota on small cell lung cancer (A-O). (A) Poseidoniaceae abundance in stool; (B) Mycobacteriaceae abundance in stool; (C) Clostridium I abundance in stool; (D) SM23-33 abundance in stool; (E) Dorea abundance in stool; (F) Bacteroides A abundance in stool; (G) Bifidobacterium longum abundance in stool; (H) CAG-349 abundance in stool; (I) UBA6398 abundance in stool; (J) Coprobacillus abundance in stool; (K) KLE1615 sp900066985 abundance in stool; (L) KLE1615 abundance in stool; (M) CAG-145 abundance in stool; (N) UBA737 sp002451855 abundance in stool; (O) Stappia abundance in stool.

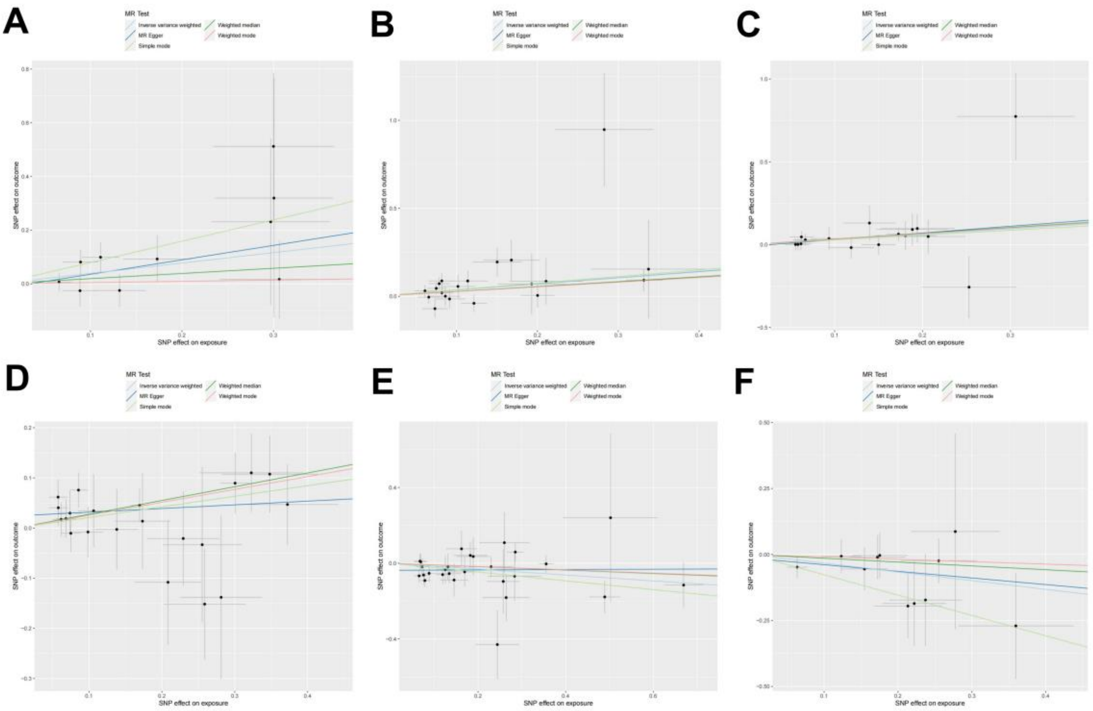

Supplementary figure 13. Scatter plot of MR analysis for 6 cytokines on lung squamous cell carcinoma (A-F). (A) Interleukin-24 levels (IL-24); (B) Eotaxin levels (CCL11); (C) Protein S100-A12 levels (EN-RAGE); (D) Monocyte chemoattractant protein-1 levels (CCL8); (E) TNF-related apoptosis-inducing ligand levels (TRAIL); (F) Interleukin-20 levels (IL-20).

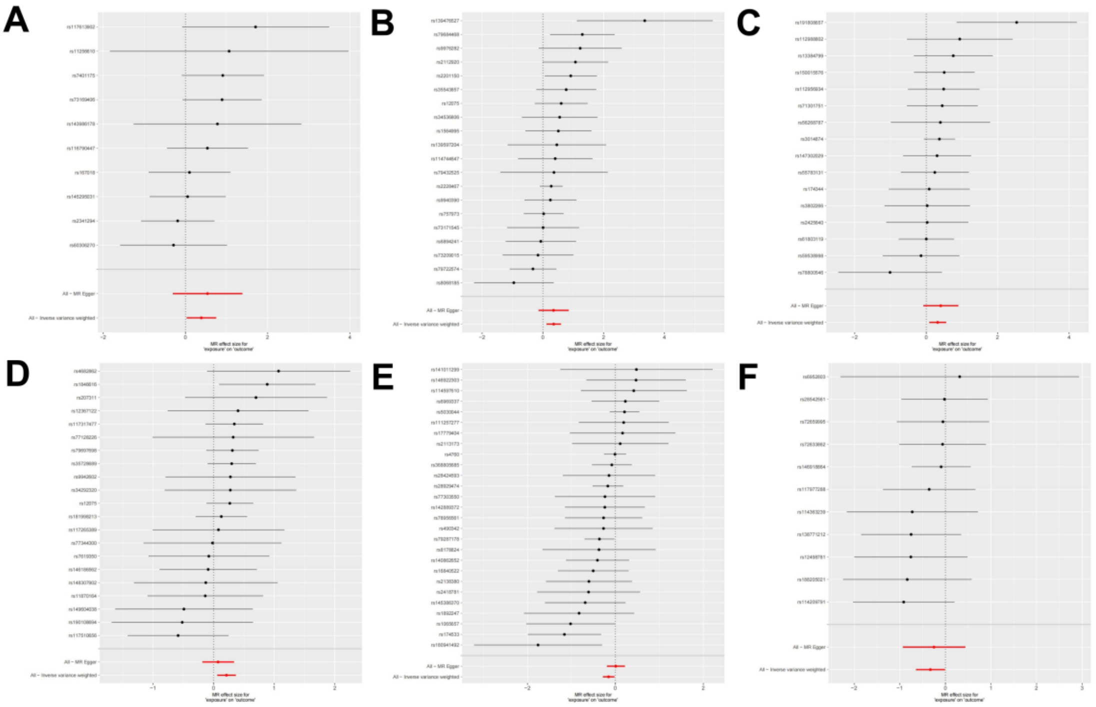

Supplementary figure 14. Forest plot of MR analysis results for 6 cytokines on lung squamous cell carcinoma (A-F). (A) Interleukin-24 levels (IL-24); (B) Eotaxin levels (CCL11); (C) Protein S100-A12 levels (EN-RAGE); (D) Monocyte chemoattractant protein-1 levels (CCL8); (E) TNF-related apoptosis-inducing ligand levels (TRAIL); (F) Interleukin-20 levels (IL-20).

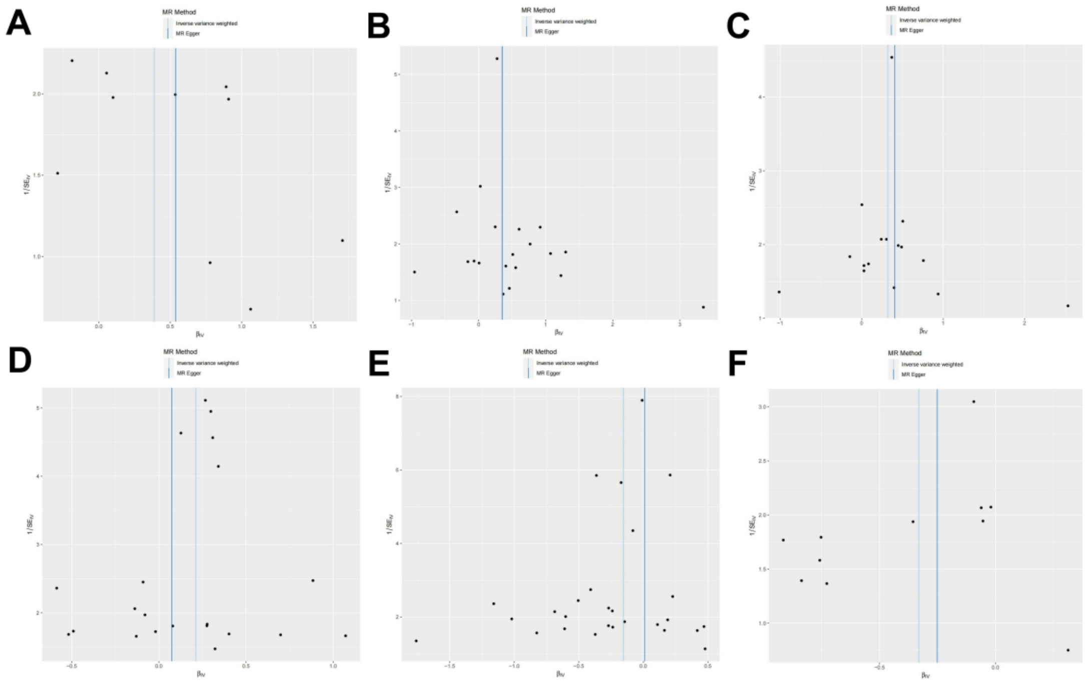

Supplementary figure 15. Funnel plot of MR analysis for 6 cytokines on lung squamous cell carcinoma (A-F). (A) Interleukin-24 levels (IL-24); (B) Eotaxin levels (CCL11); (C) Protein S100-A12 levels (EN-RAGE); (D) Monocyte chemoattractant protein-1 levels (CCL8); (E) TNF-related apoptosis-inducing ligand levels (TRAIL); (F) Interleukin-20 levels (IL-20).

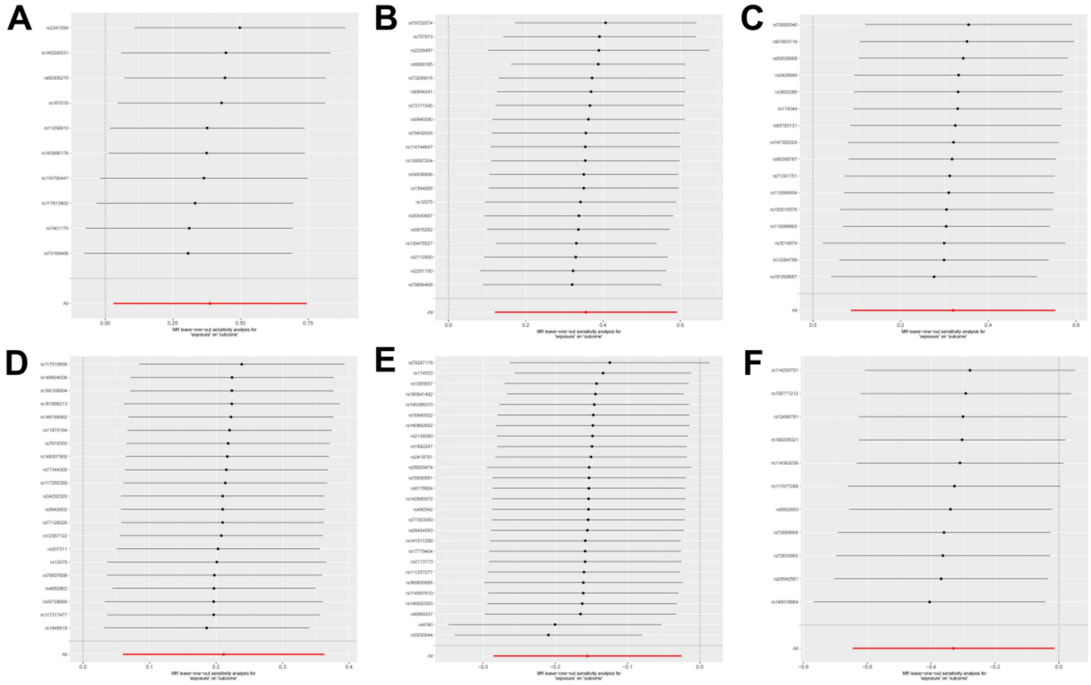

Supplementary figure 16. Leave-one-out plot of MR analysis for 6 cytokines on lung squamous cell carcinoma (A-F). (A) Interleukin-24 levels (IL-24); (B) Eotaxin levels (CCL11); (C) Protein S100-A12 levels (EN-RAGE); (D) Monocyte chemoattractant protein-1 levels (CCL8); (E) TNF-related apoptosis-inducing ligand levels (TRAIL); (F) Interleukin-20 levels (IL-20).

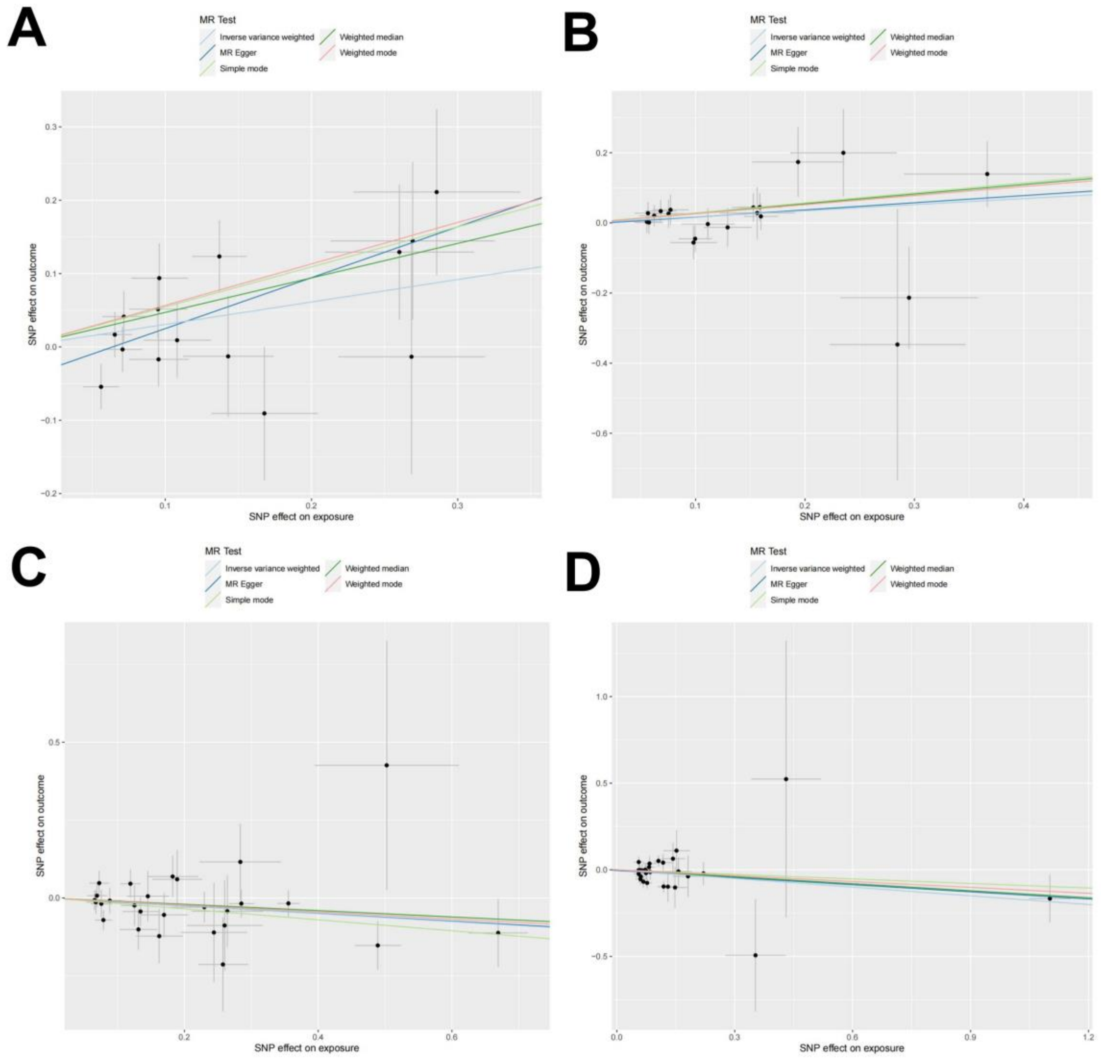

Supplementary figure 17. Scatter plot of MR analysis for 4 cytokines on lung adenocarcinoma (A-D). (A) Transforming growth factor-alpha levels (TGF-alpha); (B) Interleukin-10 levels (IL-10); (C) TNF-related apoptosis-inducing ligand levels (TRAIL); (D) Fms-related tyrosine kinase 3 ligand levels (FIt3L).

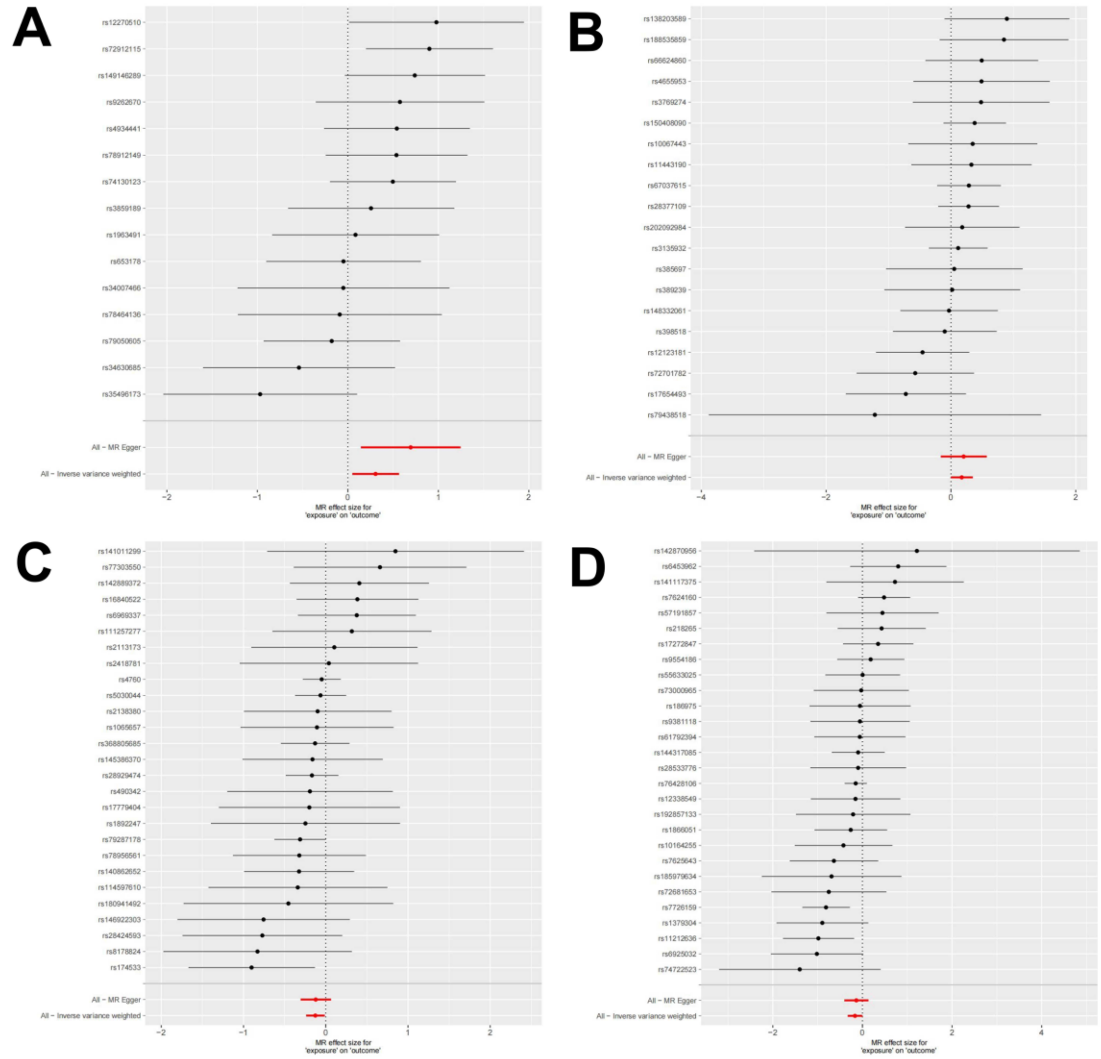

Supplementary figure 18. Forest plot of MR analysis results for 4 cytokines on lung adenocarcinoma (A-D). (A) Transforming growth factor-alpha levels (TGF-alpha); (B) Interleukin-10 levels (IL-10); (C) TNF-related apoptosis-inducing ligand levels (TRAIL); (D) Fms-related tyrosine kinase 3 ligand levels (FIt3L).

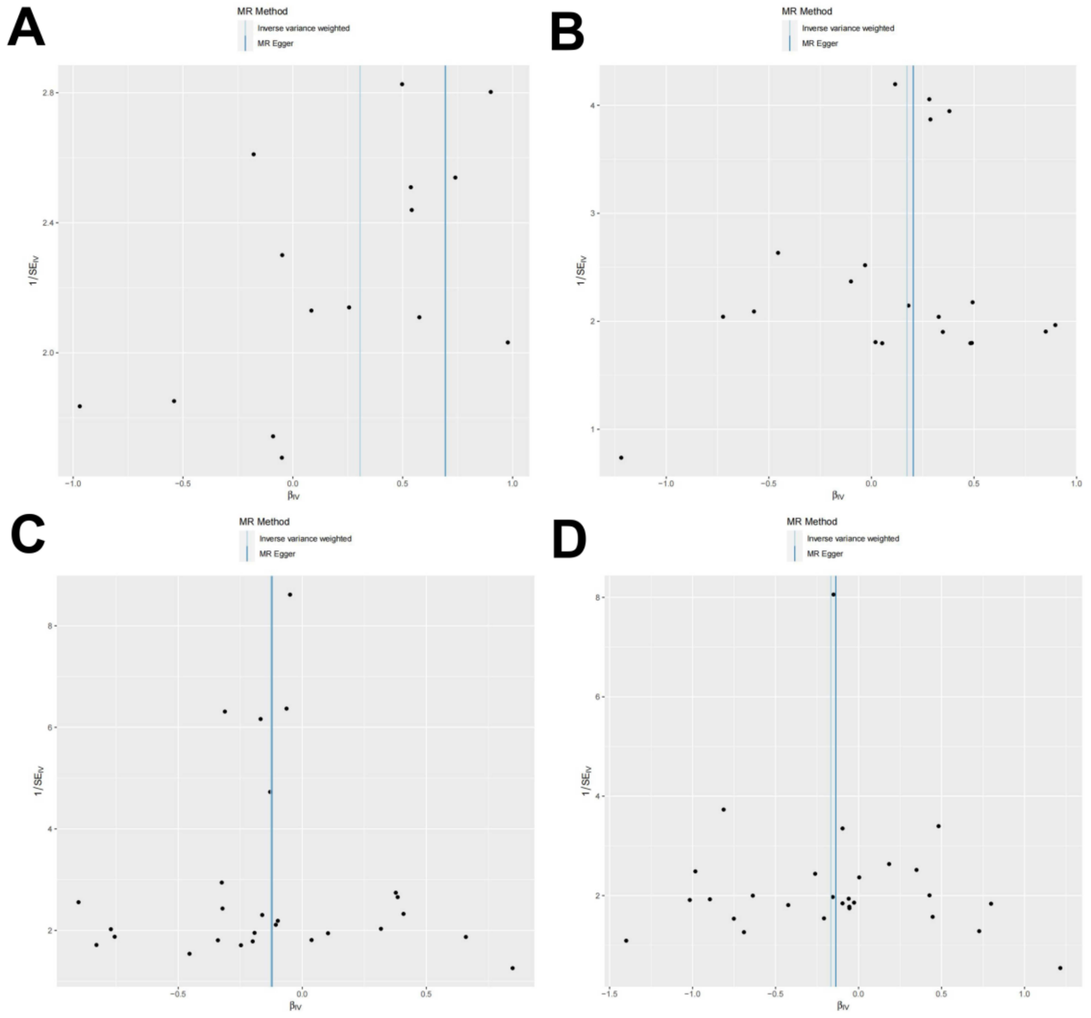

Supplementary figure 19. Funnel plot of MR analysis for 4 cytokines on lung adenocarcinoma (A-D). (A) Transforming growth factor-alpha levels (TGF-alpha); (B) Interleukin-10 levels (IL-10); (C) TNF-related apoptosis-inducing ligand levels (TRAIL); (D) Fms-related tyrosine kinase 3 ligand levels (FIt3L).

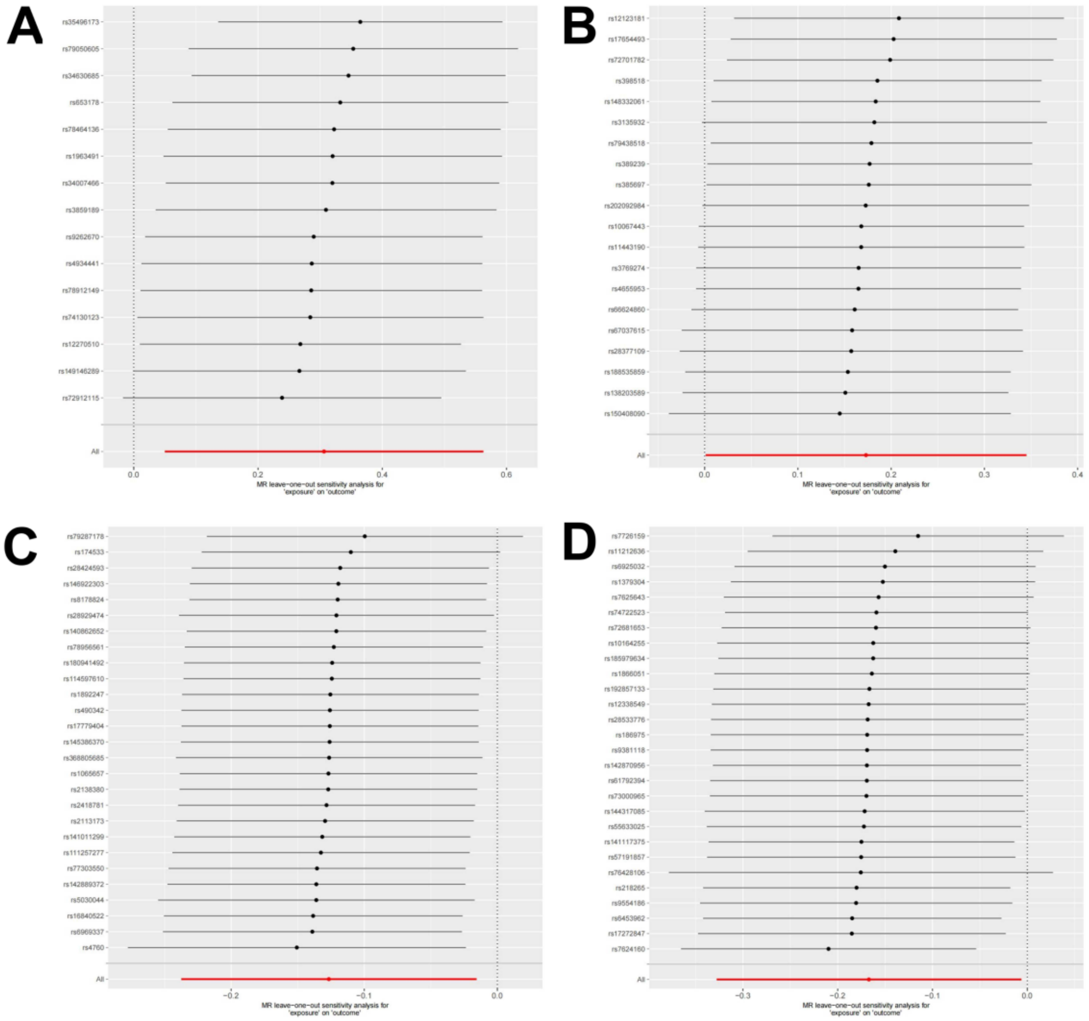

Supplementary figure 20. Leave-one-out plot of MR analysis for 4 cytokines on lung adenocarcinoma (A-D). (A) Transforming growth factor-alpha levels (TGF-alpha); (B) Interleukin-10 levels (IL-10); (C) TNF-related apoptosis-inducing ligand levels (TRAIL); (D) Fms-related tyrosine kinase 3 ligand levels (FIt3L).

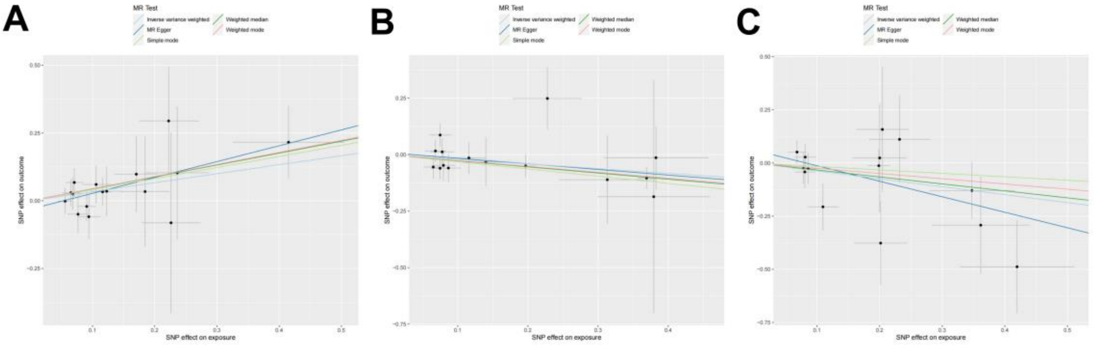

Supplementary figure 21. Scatter plot of MR analysis for 3 cytokines on small cell lung cancer (A-C). (A) Fibroblast growth factor 23 levels (FGF-23); (B) Interleukin-15 receptor subunit alpha levels (IL-15RA); (C) Caspase 8 levels (CASP-8).

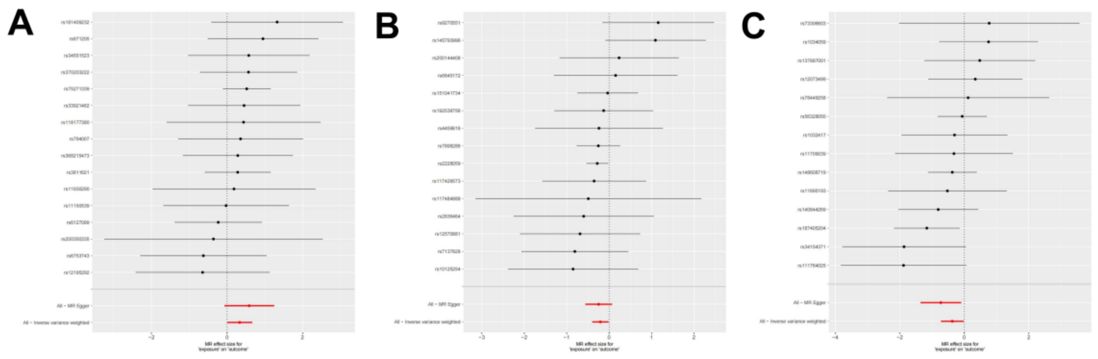

Supplementary figure 22. Forest plot of MR analysis results for 3 cytokines on small cell lung cancer (A-C). (A) Fibroblast growth factor 23 levels (FGF-23); (B) Interleukin-15 receptor subunit alpha levels (IL-15RA); (C) Caspase 8 levels (CASP-8).

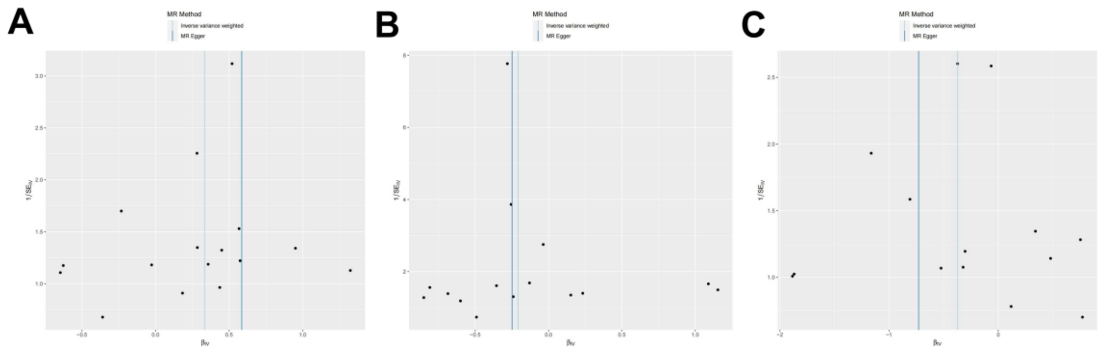

Supplementary figure 23. Funnel plot of MR analysis for 3 cytokines on small cell lung cancer (A-C). (A) Fibroblast growth factor 23 levels (FGF-23); (B) Interleukin-15 receptor subunit alpha levels (IL-15RA); (C) Caspase 8 levels (CASP-8).

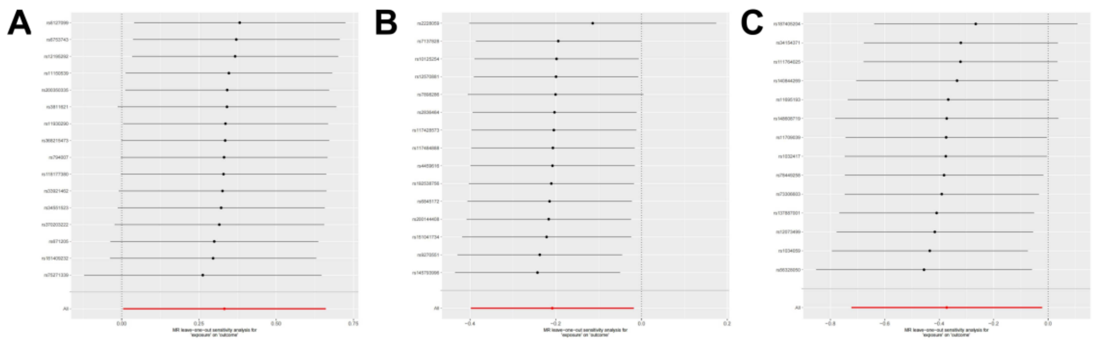

Supplementary figure 24. Leave-one-out plot of MR analysis for 3 cytokines on small cell lung cancer (A-C). (A) Fibroblast growth factor 23 levels (FGF-23); (B) Interleukin-15 receptor subunit alpha levels (IL-15RA); (C) Caspase 8 levels (CASP-8).

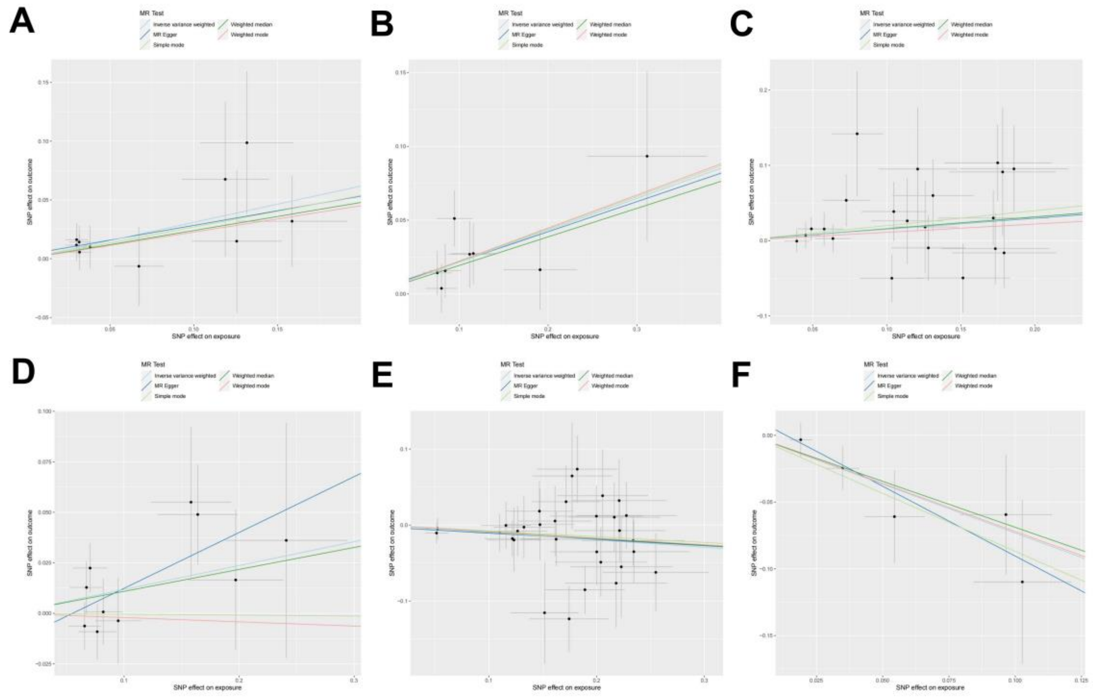

Supplementary figure 25. Scatter plot of MR analysis for gut microbiota on cytokines (A-F). (A) Atopobiaceae abundance in stool versus Interleukin-24 levels (IL-24); (B) Faecalicatena lactaris abundance in stool versus Interleukin-20 levels (IL-20); (C) CAG-632 abundance in stool versus Interleukin-20 levels (IL-20); (D) Veillonellaceae abundance in stool versus Monocyte chemoattractant protein-1 levels (CCL8); (E) Megamonas abundance in stool versus Interleukin-24 levels (IL-24); (F) Geobacteraceae abundance in stool versus Eotaxin levels (CCL11).

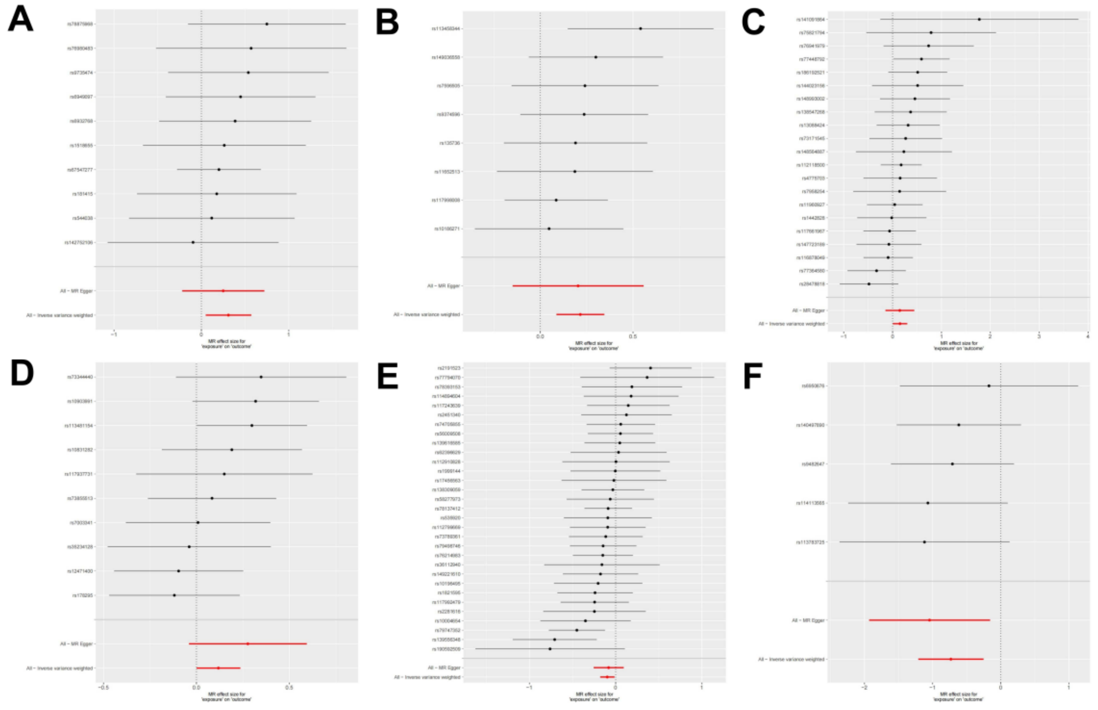

Supplementary figure 26. Forest plot of MR analysis results for gut microbiota on cytokines (A-F). (A) Atopobiaceae abundance in stool versus Interleukin-24 levels (IL-24); (B) Faecalicatena lactaris abundance in stool versus Interleukin-20 levels (IL-20); (C) CAG-632 abundance in stool versus Interleukin-20 levels (IL-20); (D) Veillonellaceae abundance in stool versus Monocyte chemoattractant protein-1 levels (CCL8); (E) Megamonas abundance in stool versus Interleukin-24 levels (IL-24); (F) Geobacteraceae abundance in stool versus Eotaxin levels (CCL11).

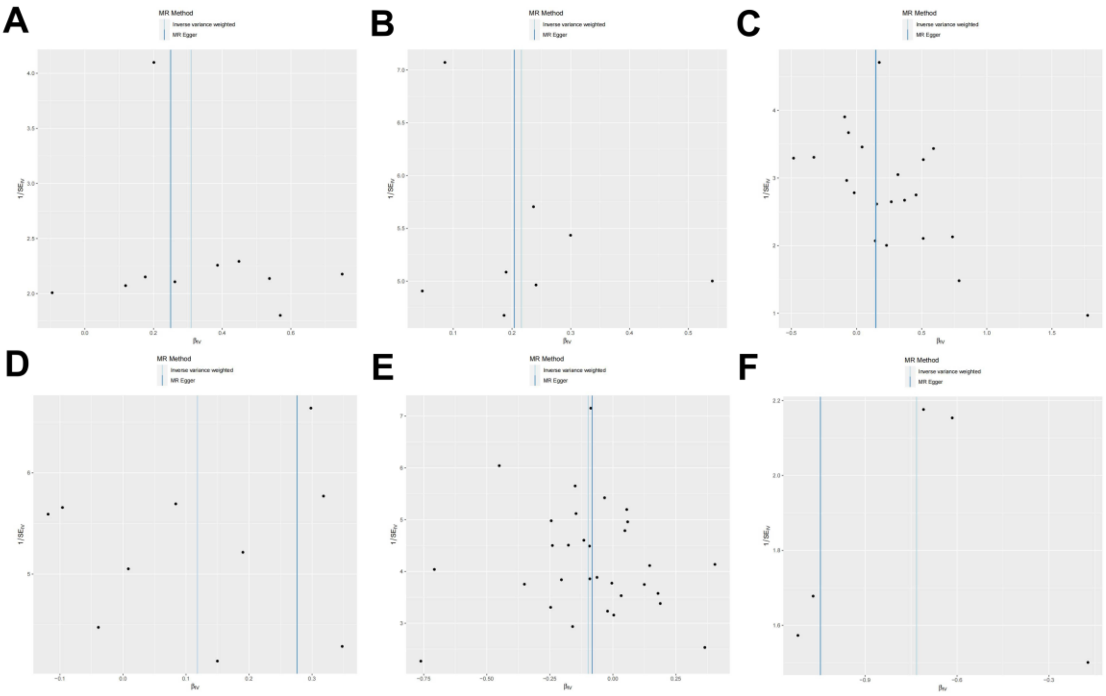

Supplementary figure 27. Funnel plot of MR analysis for gut microbiota on cytokines (A-F). (A) Atopobiaceae abundance in stool versus Interleukin-24 levels (IL-24); (B) Faecalicatena lactaris abundance in stool versus Interleukin-20 levels (IL-20); (C) CAG-632 abundance in stool versus Interleukin-20 levels (IL-20); (D) Veillonellaceae abundance in stool versus Monocyte chemoattractant protein-1 levels (CCL8); (E) Megamonas abundance in stool versus Interleukin-24 levels (IL-24); (F) Geobacteraceae abundance in stool versus Eotaxin levels (CCL11).

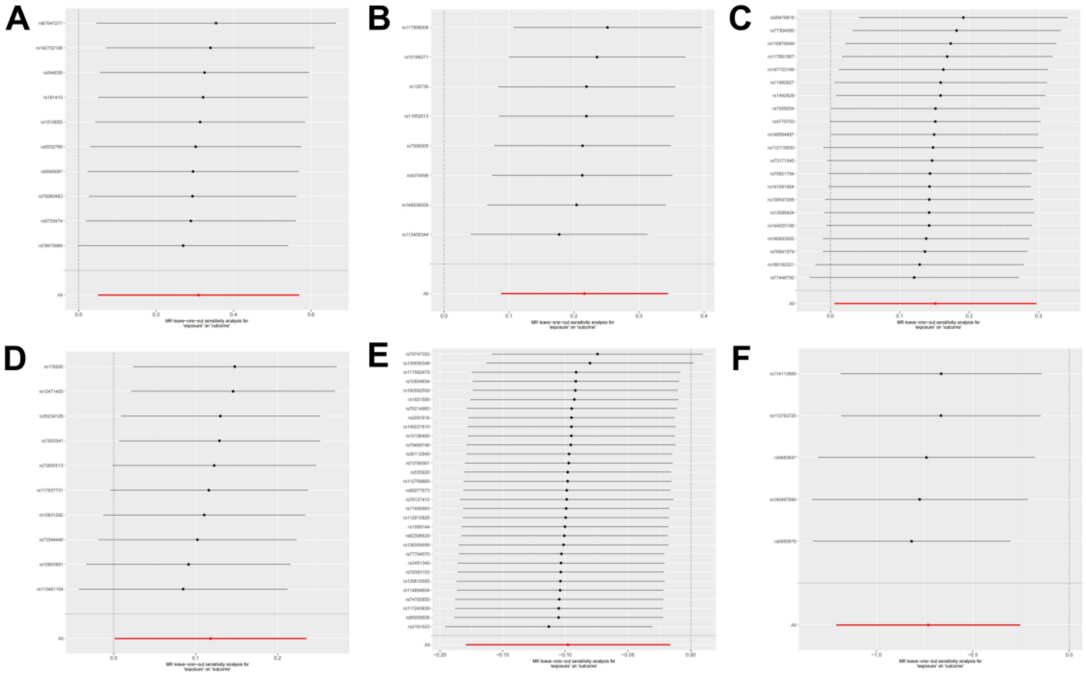

Supplementary figure 28. Leave-one-out plot of MR analysis for gut microbiota on cytokines (A-F). (A) Atopobiaceae abundance in stool versus Interleukin-24 levels (IL-24); (B) Faecalicatena lactaris abundance in stool versus Interleukin-20 levels (IL-20); (C) CAG-632 abundance in stool versus Interleukin-20 levels (IL-20); (D) Veillonellaceae abundance in stool versus Monocyte chemoattractant protein-1 levels (CCL8); (E) Megamonas abundance in stool versus Interleukin-24 levels (IL-24); (F) Geobacteraceae abundance in stool versus Eotaxin levels (CCL11).

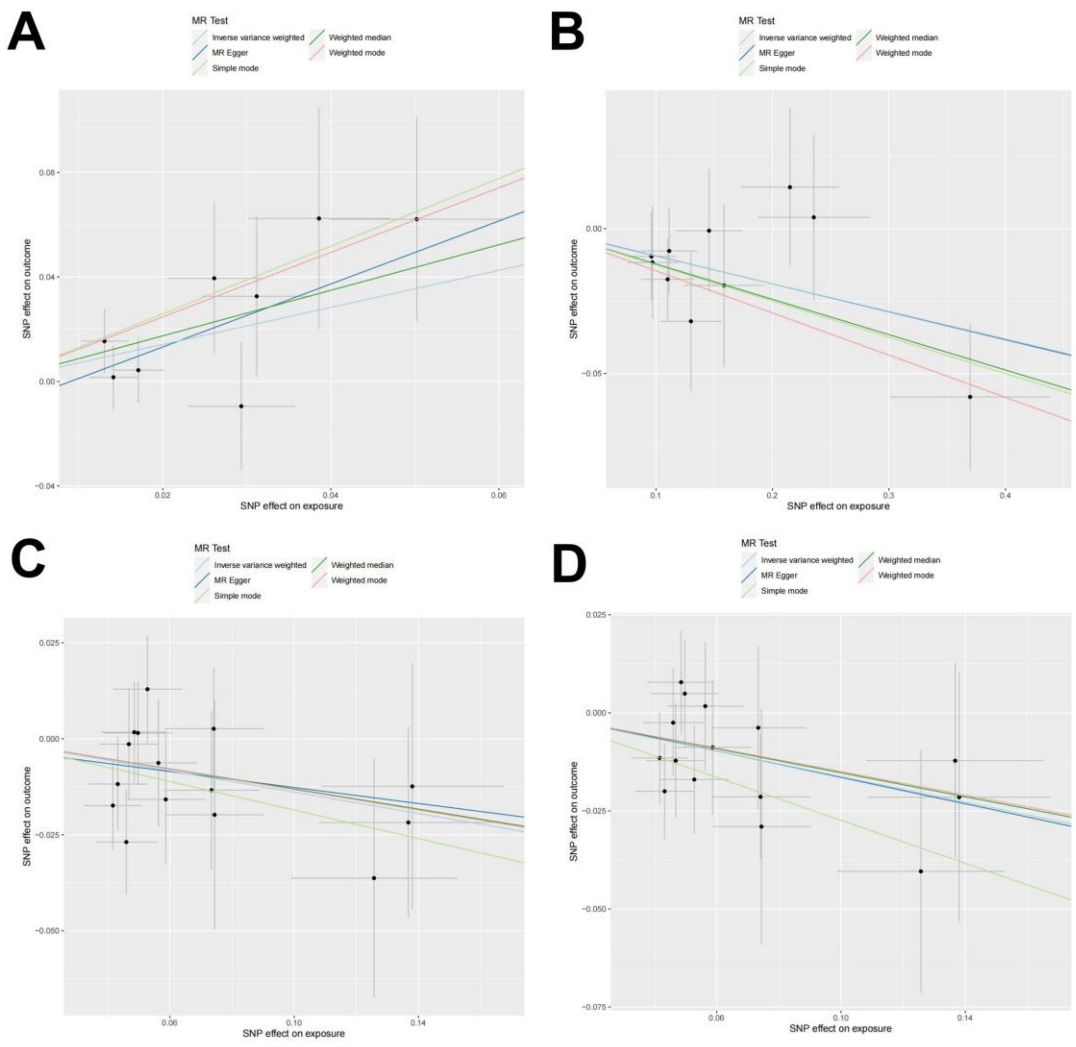

Supplementary figure 29. Scatter plot of MR analysis for gut microbiota on cytokines (A-D). (A) Bacillaceae A abundance in stool versus Fms-related tyrosine kinase 3 ligand levels (FIt3L); (B) Victivallis sp002998355 abundance in stool versus TNF-related apoptosis-inducing ligand levels (TRAIL); (C) GCA-900066495 sp900066495 abundance in stool versus TNF-related apoptosis-inducing ligand levels (TRAIL); (D) GCA-900066495 sp900066495 abundance in stool versus Transforming growth factor-alpha levels (TGF-alpha).

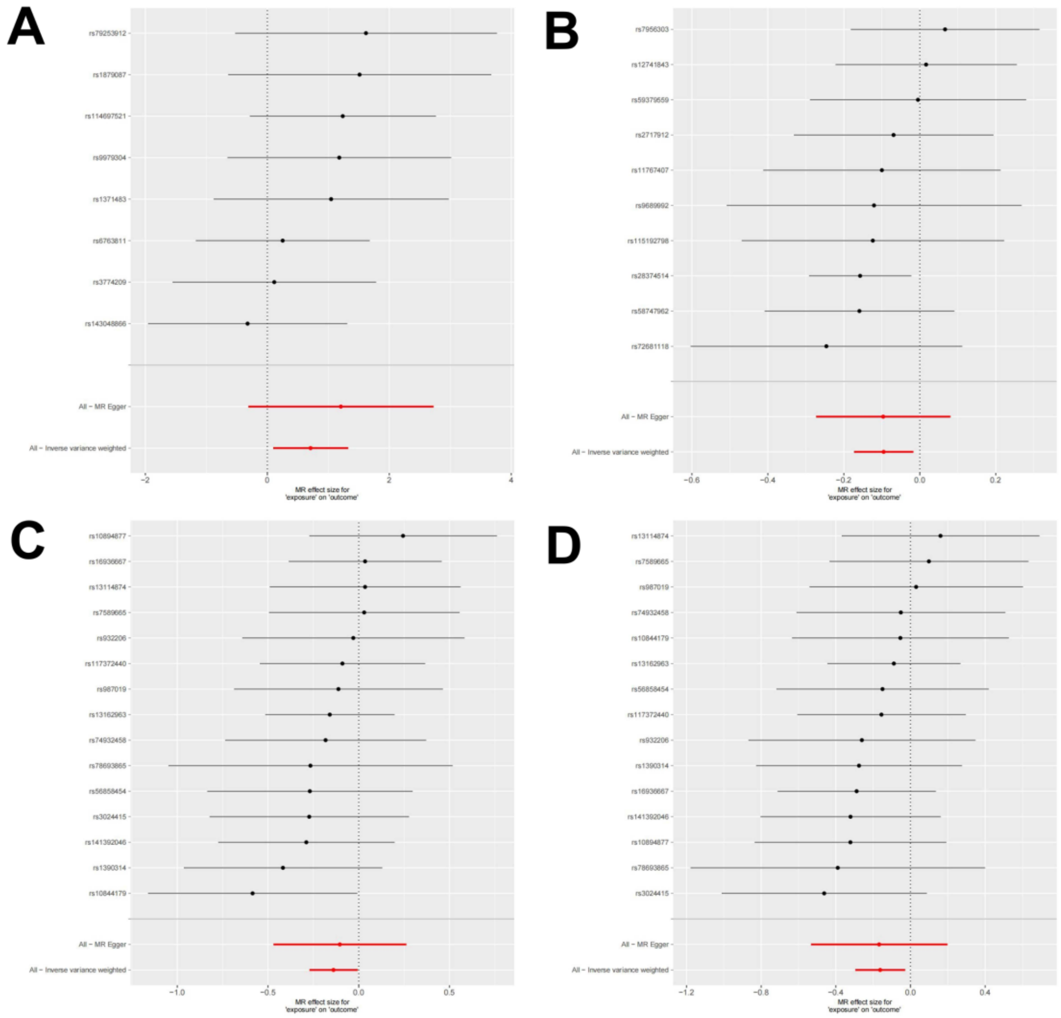

Supplementary figure 30. Forest plot of MR analysis results for gut microbiota on cytokines (A-D). (A) Bacillaceae A abundance in stool versus Fms-related tyrosine kinase 3 ligand levels (FIt3L); (B) Victivallis sp002998355 abundance in stool versus TNF-related apoptosis-inducing ligand levels (TRAIL); (C) GCA-900066495 sp900066495 abundance in stool versus TNF-related apoptosis-inducing ligand levels (TRAIL); (D) GCA-900066495 sp900066495 abundance in stool versus Transforming growth factor-alpha levels (TGF-alpha).

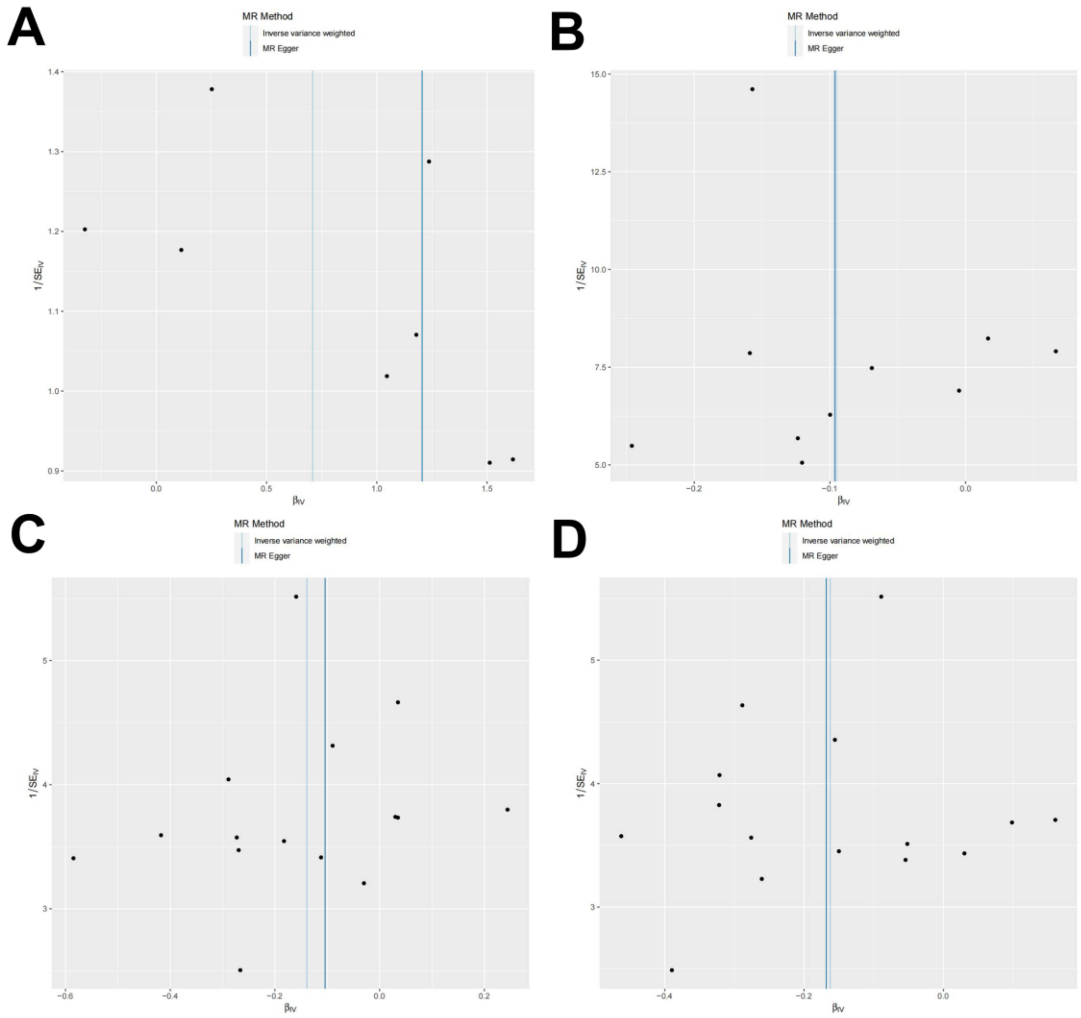

Supplementary figure 31. Funnel plot of MR analysis for gut microbiota on cytokines (A-D). (A) Bacillaceae A abundance in stool versus Fms-related tyrosine kinase 3 ligand levels (FIt3L); (B) Victivallis sp002998355 abundance in stool versus TNF-related apoptosis-inducing ligand levels (TRAIL); (C) GCA-900066495 sp900066495 abundance in stool versus TNF-related apoptosis-inducing ligand levels (TRAIL); (D) GCA-900066495 sp900066495 abundance in stool versus Transforming growth factor-alpha levels (TGF-alpha).

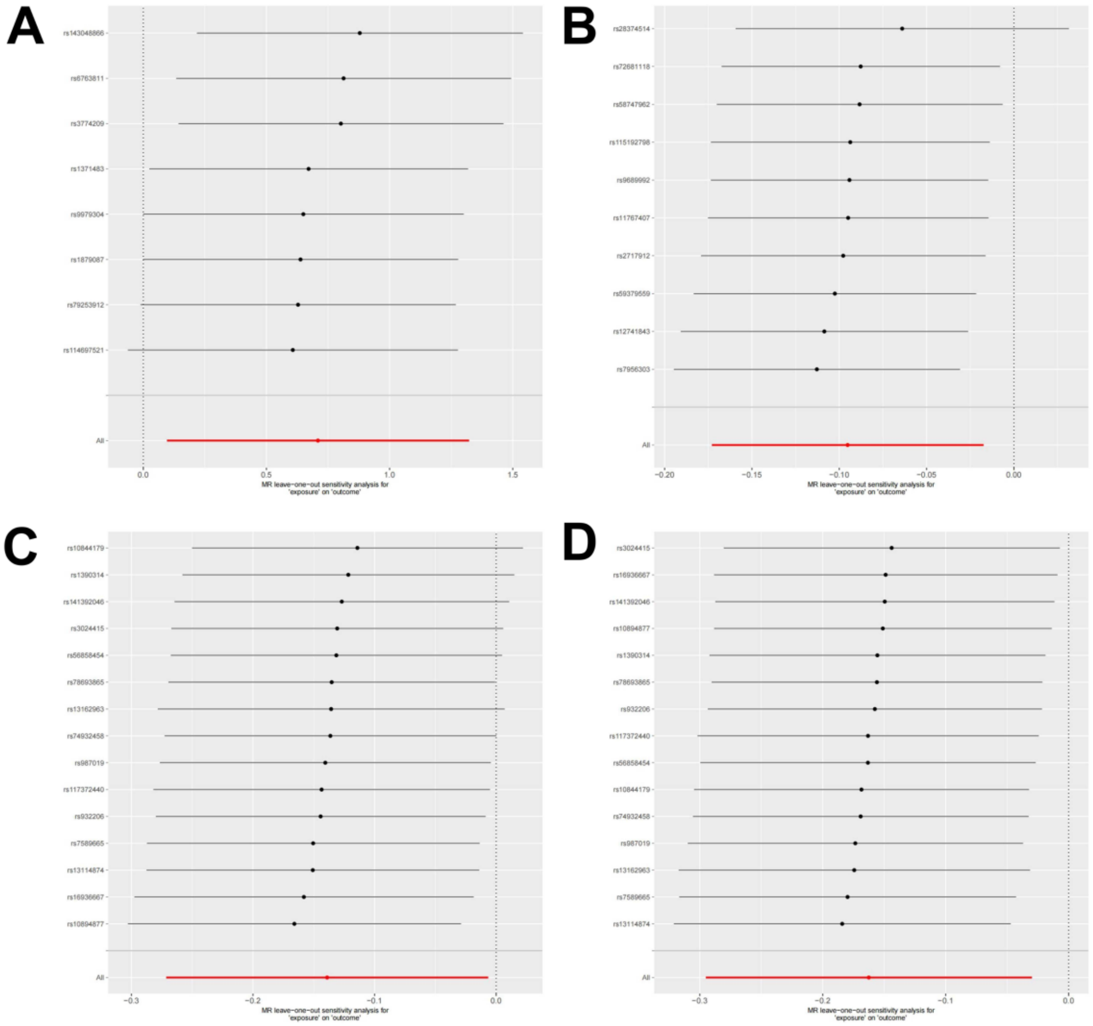

Supplementary figure 32. Leave-one-out plot of MR analysis for gut microbiota on cytokines (A-D). (A) Bacillaceae A abundance in stool versus Fms-related tyrosine kinase 3 ligand levels (FIt3L); (B) Victivallis sp002998355 abundance in stool versus TNF-related apoptosis-inducing ligand levels (TRAIL); (C) GCA-900066495 sp900066495 abundance in stool versus TNF-related apoptosis-inducing ligand levels (TRAIL); (D) GCA-900066495 sp900066495 abundance in stool versus Transforming growth factor-alpha levels (TGF-alpha).

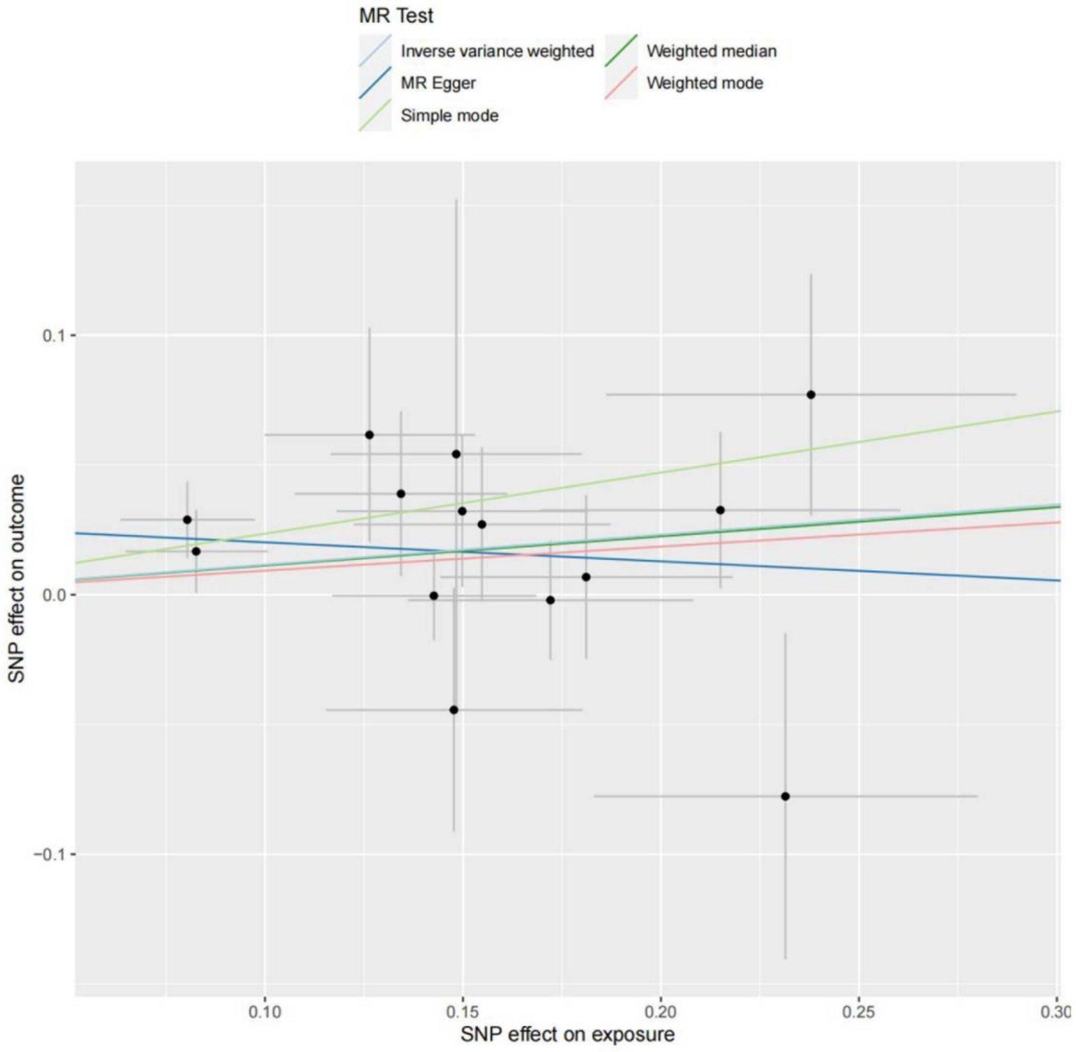

Supplementary figure 33. Scatter plot of MR analysis for gut microbiota on cytokines (UBA6398 abundance in stool versus Fibroblast growth factor 23 levels).

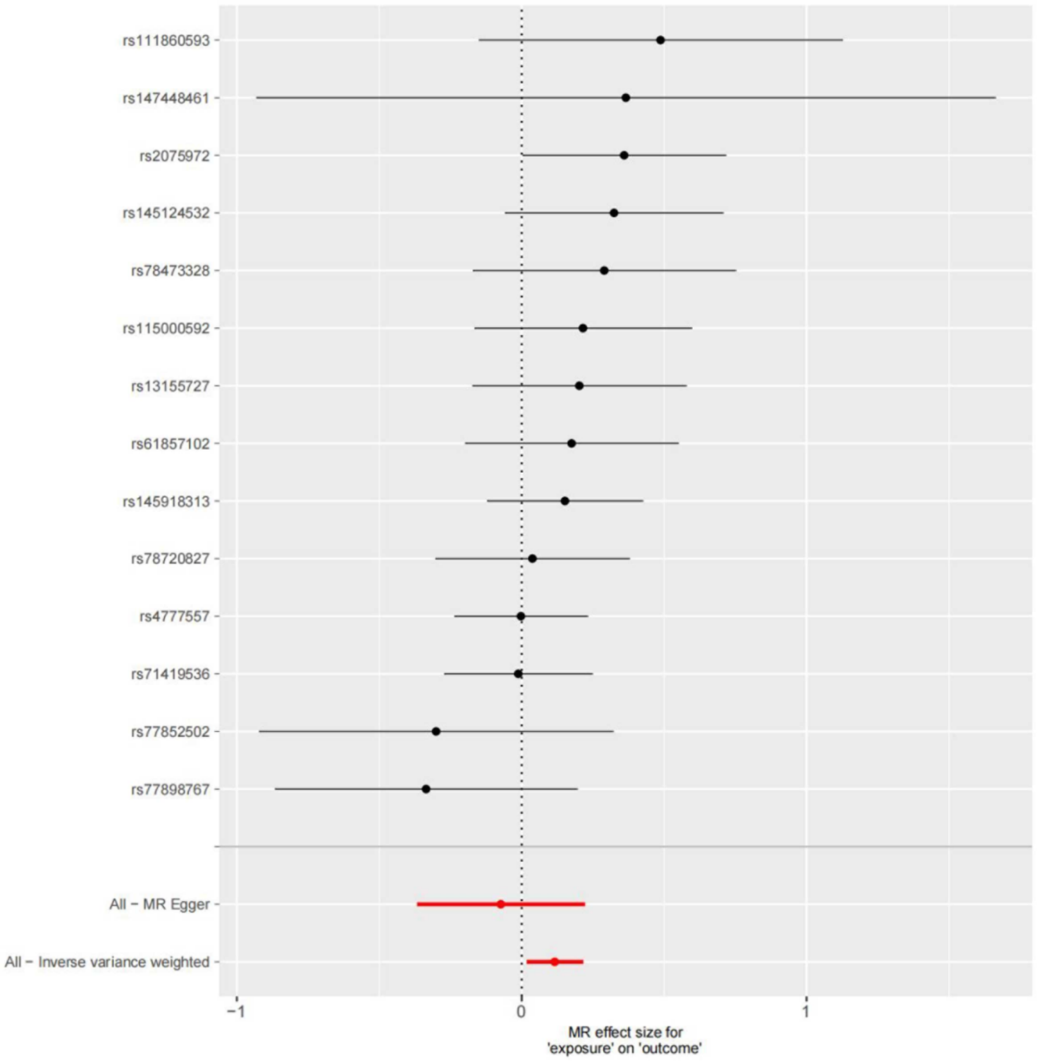

Supplementary figure 34. Forest plot of MR analysis results for gut microbiota on cytokines (UBA6398 abundance in stool versus Fibroblast growth factor 23 levels).

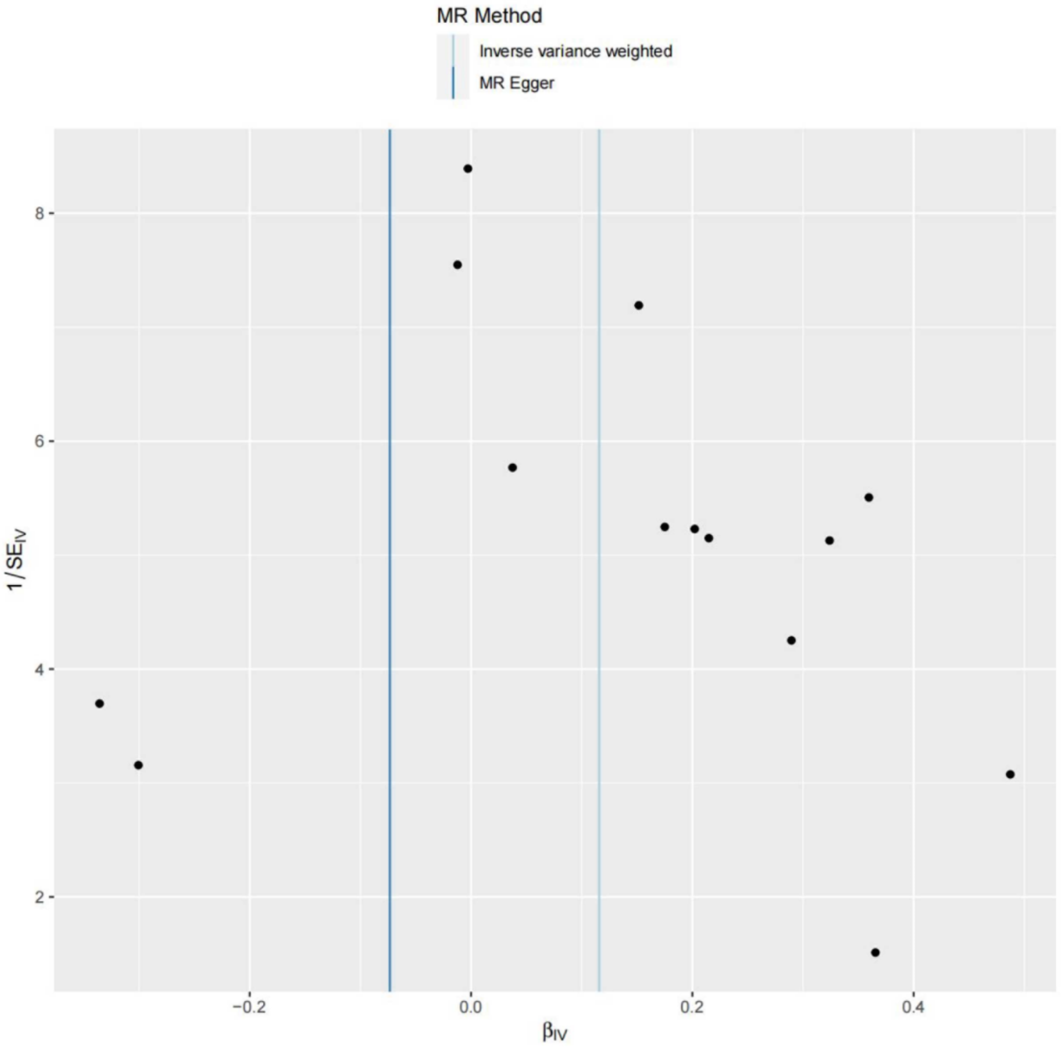

Supplementary figure 35. Funnel plot of MR analysis for gut microbiota on cytokines (UBA6398 abundance in stool versus Fibroblast growth factor 23 levels).

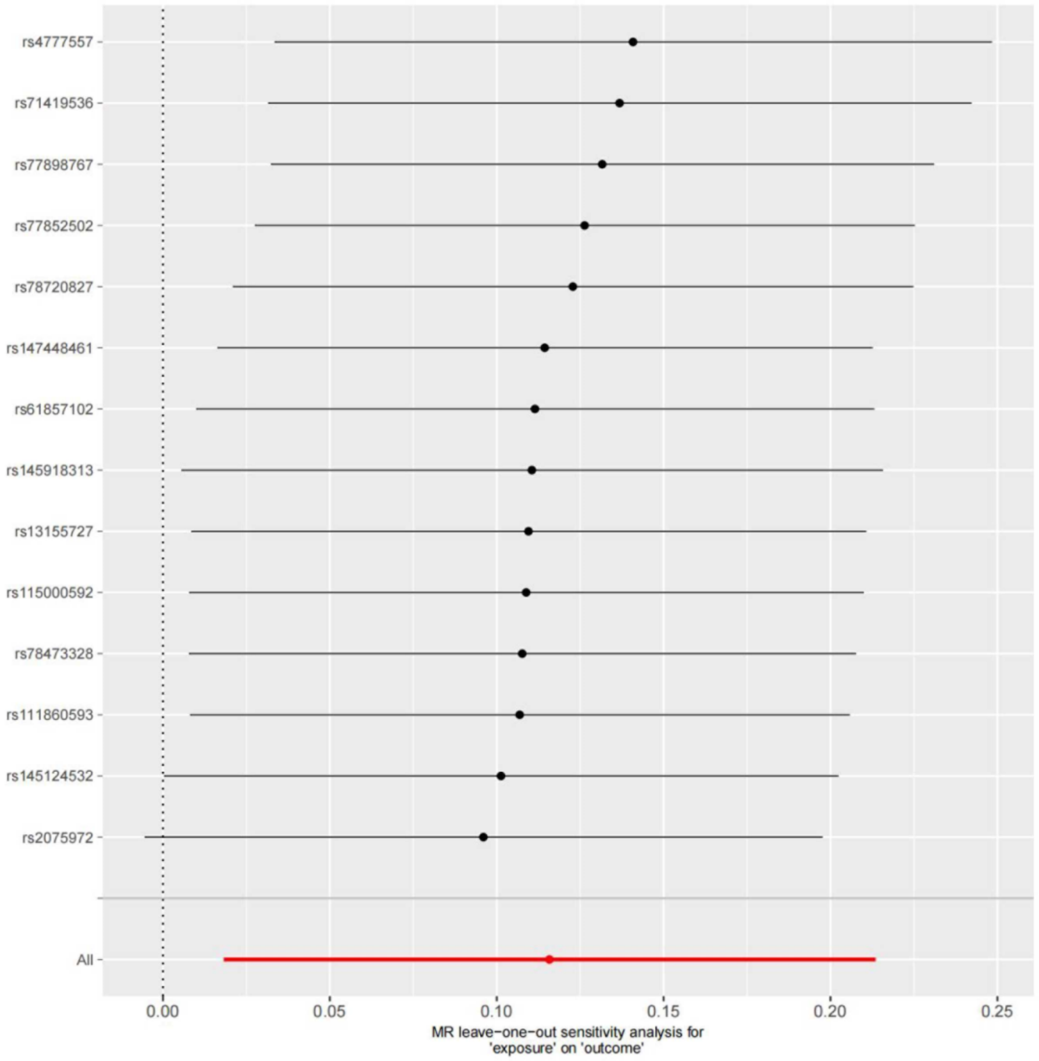

Supplementary figure 36. Leave-one-out plot of MR analysis for gut microbiota on cytokines (UBA6398 abundance in stool versus Fibroblast growth factor 23 levels).
